# Unlocking the Impact: A Systematic Review and Meta-Analysis of Biomechanical Insights into Rugby Head Impacts Using Wearable Sensor Technology

**DOI:** 10.1007/s40279-025-02228-z

**Published:** 2025-05-03

**Authors:** Luis De Sousa-De Sousa, Hugo G. Espinosa, José Luis Maté-Muñoz, Roberto Murias-Lozano, Mario Iglesias Muñiz, Francisco Javier San Sebastián Obregón, Cristian Solís-Mencía, Pablo García-Fernández

**Affiliations:** 1 Department of Radiology, Rehabilitation and Physiotherapy, Faculty of Nursing, Physiotherapy and Podiatry, Complutense University of Madrid, 28040 Madrid, Spain; 2 School of Engineering and Built Environment, Griffith University, Brisbane, QLD 4111 Australia; 3 Department of Physiotherapy, Camilo José Cela University, Villafranca del Castillo, 28692 Madrid, Spain; 4 Centro Médico-Quirúrgico Olympia, P.º de la Castellana, 259, Fuencarral-El Pardo, 28046 Madrid, Spain; 5 Spanish Rugby Federation, 28008 Madrid, Spain; 6 Department of Medicine, Faculty of Health Sciences, University of Deusto, Bilbao, Bizkaia Spain; 7 Grupo InPhysio, Instituto de Investigación Sanitaria del Hospital Clínico San Carlos (IdISSC), Madrid, Spain

## Abstract

**Background:**

In the realm of sports medicine, understanding the biomechanics of head impacts, particularly in contact sports such as rugby, is of utmost interest for injury prevention and player safety.

**Objective:**

This systematic review and meta-analysis aims to consolidate existing knowledge on head impacts in rugby using wearable sensor technology, focusing on peak linear acceleration, peak rotational acceleration, and impact location.

**Methods:**

A systematic search of electronic databases [PubMed, Web of Science (WOS), Scopus, Embase, SPORTDiscus, PsycINFO, and CINAHL] was conducted in March 2024, including studies that assessed head impacts with wearable technology in rugby athletes. The search did not impose any restrictions on publication dates and included studies published in English and Spanish. A random-effects meta-analysis model was employed to combine comparable data from the included studies.

**Results:**

The literature search yielded 13 prospective cohort studies, collectively analyzing 895 participants and 44,036 head impacts. Most studies were conducted in Australasia and North America, with varying levels of play represented, from junior to semi/professional and from both rugby codes, rugby union (RU) and rugby league (RL). Wearable sensors, including instrumented mouthguards and skin patches, were utilized to measure head impact kinematics, with peak linear acceleration consistently reported across all studies. Results reveal significant heterogeneity in peak linear and rotational acceleration, highlighting the complexity of quantifying impact magnitudes in rugby. Impact location analysis indicated side impacts as most prevalent (44%), followed by frontal (29%) and back impacts (19%). Notably, concussive events yielded a pooled peak linear acceleration estimate of 63.01 g, with the RL cohort exhibiting higher acceleration than RU.

**Conclusion:**

This study contributes to the growing body of literature on head impacts in rugby; identifying available evidence on the magnitude and location of head impacts measured by sensors, and emphasizing the importance of wearable sensor technology in advancing player safety and informing injury management practices. Despite the valuable insights provided, limitations, including methodological inconsistencies and study heterogeneity, underscore the need for cautious interpretation. Further research is warranted to standardize protocols and enhance the understanding of effective injury prevention strategies in rugby. PROSPERO registration number: CRD42023480779 (20 November 2023).

**Supplementary Information:**

The online version contains supplementary material available at 10.1007/s40279-025-02228-z.

## Key Points

**Table:** 

This systematic review aimed to identify the kinematics and locations of head impacts, along with concussive events, recorded by inertial sensors among rugby players from rugby union and rugby league.
Head impacts mean kinematics were 17.35 *g* (95% CI 14.68–20.02 *g*) for rugby union peak linear acceleration and 25.19 *g* (95% CI 7.64–42.73 *g*) for rugby league, and 1259.51 rad/s^2^ (95% CI 1112.12–1406.91 rad/s^2^) for rugby union peak rotational acceleration.
Distribution of impact location was 44% on the side of the head (95% CI 28–60%), 29% on the front (95% CI 22–37%), 19% on the back (95% CI 12–25%), and 7% on the top of the head (95% CI 1–13%).
Concussive events mean peak linear acceleration was 63.01 *g* (95% CI 45.53–80.49 *g*).
The findings both synthesize and analyze existing literature, highlighting the importance of caution when interpreting the data. Considerations include limited literature availability, sensor technology variations, methodological differences, and the ongoing evolution of sensor technology.

## Introduction

In recent years, the exploration of biomechanical aspects related to head impacts in sports, particularly in rugby, has gained considerable traction [1]. Central to this inquiry is understanding (and identifying potential ways to reduce) the number and severity of sports-related concussions (SRC), a prevalent and potentially serious form of traumatic brain injury [2]. SRC is defined as an injury caused by biomechanical forces, often from a direct blow to the head or body, resulting in an impulsive force being transmitted to the brain that occurs in sports and exercise-related activities. This initiates a neurotransmitter and metabolic cascade, with possible axonal injury, blood flow change, and inflammation affecting the brain. Symptoms and signs may present immediately or evolve over minutes or hours and commonly resolve within days but may be prolonged. No abnormality is seen in standard structural neuroimaging studies, but in the research setting, abnormalities may be present in functional, blood flow, or metabolic imaging studies [3]. Despite their prevalence, concussions present challenges in diagnosis, and measuring head impacts helps assess the risk of potential concussions and implement timely interventions when necessary [3, 4].

The advent of wearable sensor technology, notably inertial sensors, has driven a paradigm shift in the study of head impacts, offering unprecedented opportunities for data collection and analysis [5]. Wearable sensors provide real-time data on motion and acceleration. They report impact biomechanics through accelerometers and gyroscopes as peak linear acceleration (PLA) measured in gravitation (*g*), and peak rotational acceleration (PRA) measured in radians per second squared (rad/s^2^) [5]. This enables researchers to quantify and analyze the biomechanics of head impacts with greater precision than previously [6].

Sensors have been widely employed to measure head impacts in various sports and activities [7]. This technological advancement holds immense promise for enhancing player safety and informing injury prevention strategies in contact sports such as rugby [8]. Notwithstanding, challenges persist in accurately assessing these impacts, for example, in their body placement, given that the placement of sensors on different parts of the body, through headbands, skin patches, or mouthguards, can lead to variations in the measured impact forces and directions [9]. Another challenge lies in the accuracy of the sensors themselves, with differing levels of precision, recording thresholds, and reliability among sensor types [9, 10]. In addition, sensors may not fully capture the complexity of head movement during impact, primarily measuring kinematics from a location different to the head’s gravitational center. Consequently, adjustments are necessary to translate sensor data into approximations of head forces [11]. In that sense, video verification can be utilized alongside inertial sensors to validate data accuracy and provide additional context for interpretation [12, 13]. This combined approach offers a more comprehensive understanding of head impact dynamics, reducing the likelihood of false positives and improving measurement accuracy [14].

Despite the challenges inherent in accurately assessing head impacts and the subsequent difficulty in integrating them, researchers have conducted high-quality systematic reviews and meta-analyses utilizing wearable sensor technology to analyze head impacts and acceleration forces in multiple sports, including American Football [15], soccer [16], and combat sports [17]. Studies of this nature have contributed valuable insights into injury mechanisms, risk factors, and patterns associated with head impacts, thereby informing injury prevention strategies and potential player safety protocols [18].

However, despite the wealth of research in other sports, the field of rugby has yet to benefit fully from similar investigations owing to the novelty of assessing head impacts through inertial sensor technology and its rapid expansion over the last 10 years [1]. This underscores the critical need for a systematic review and meta-analysis to consolidate existing knowledge and bridge the gap in research pertaining to rugby head impacts.

The proposed meta-analysis, guided by the population, intervention, comparator, and outcome(s) (PICO) framework, seeks to address this gap by synthesizing and analyzing available literature on biomechanical insights from head impacts using sensor technology in rugby training or matches. By including observational studies encompassing participants of all ages and levels of play, this meta-analysis aims to provide a comprehensive understanding of head impact biomechanics across diverse rugby populations and an overview of the current state of knowledge in this field.

## Methods

This systematic review was conducted and reported in accordance with the Preferred Reporting Items for Systematic Reviews and Meta-Analysis (PRISMA) guidelines [19], as well as the Meta-analysis Of Observational Studies in Epidemiology (MOOSE) reporting guidelines [20]. A protocol was registered on PROSPERO (registration number CRD42023480779) and is available for access at https://www.crd.york.ac.uk/prospero/display_record.php?RecordID=480779.

### Design

A systematic literature search was conducted up to March 2024 with no date limitation on the following seven electronic databases: PubMed, Web of Science (WOS), Scopus, Embase, SPORTDiscus, PsycINFO, and CINAHL. The snowball method [21] was employed to ensure comprehensive retrieval of all existing literature on rugby head impacts assessed with wearable sensors. Efforts were made to retrieve missing information by contacting authors directly via e-mail, requesting additional details or clarification of their studies. Piloting of the search strategy was supervised and collaboratively executed with an expert librarian in health sciences from the library of the Complutense University of Madrid incorporating both controlled and natural language strategies. For an illustration of our search strategy refer to Table 1, and for the full search string for all databases refer to the supplementary documents (Online Resource 1).

**Table 1:** PubMed search string

Conducted (10 March 2024)	
Search strategy	Natural, MeSH terms, and applied equations
No. 1	“rugby, union play” OR “play rugby, union” OR “union play rugbies” OR “union play rugby” OR “rugby, league play” OR “league play rugby” OR “play rugby, league” OR “rugby union” OR “rugby”/exp OR “rugby”
No. 2	“athletic injuries” OR “concussion” OR “sports concussion” OR “sports-related concussion” OR “brain concussion” OR “brain injury” OR “brain injuries” OR “mild traumatic brain injury” OR “mtbi” OR “traumatic brain injury” OR “tbi” OR “craniocerebral trauma” OR “head injury” OR “brain damage” OR “athlet* injuri*” OR (“athlet*” AND “statistics”) OR (“athlet*” AND “numerical data”) OR (“athlet* injur*” AND “epidemiology”) OR (“athlet* injur*” AND “etiology”) OR (“athlet* injur*” AND “control”)
No. 3	“head acceleration*” OR “accelerometer” OR “gyroscope” OR “wearable sensor*” OR “wearable head sensor” OR “instrumented mouthguard” OR “headgear” OR “instrumented headgear” OR “instrumented helmet” OR “instrumented skin patch”
No. 4	No. 1 AND no. 2 AND no. 3
No. 5	“rugby”/exp AND “brain concussion”/exp AND “biomechanics”/exp AND “apparatus and instruments”/exp
No. 6	No. 4 OR no. 5
Total retrieved = 228	

### Study Inclusion Criteria

Inclusion and exclusion criteria were established before the search process. Articles were included if written in either English or Spanish and if they focused on studying rugby players while utilizing wearables to measure head-impact mechanics. Exclusion criteria encompassed laboratory studies, review papers, or commentaries, as well as those involving non-rugby samples or accelerometers not tailored for impact assessment. In addition, studies without video verification were excluded to ensure data reliability. Rotational acceleration data from X2 patch devices were also excluded from the meta-analysis owing to reliability concerns. Following these criteria facilitated the compilation of in vivo studies to evaluate and present aggregated head-impact data.

After eliminating duplicates, search results underwent independent screening by two researchers (L.D.S. and P.G.F.) against the predefined eligibility criteria. References not eliminated by title or abstract were retrieved and independently assessed by full text for inclusion. Reviewers remained unblinded to the title or authors of publications, with discrepancies resolved through discussion or consultation with a third researcher (R.M.L.). This process was performed using CADIMA online software (version 2.2.4.2 [22], www.cadima.info) following the PRISMA flow diagram. Reference lists of retrieved papers were manually searched for additional potentially eligible studies.

### Data Extraction

The data extraction process was conducted meticulously, with the main researcher responsible for extracting data from the included studies. A second investigator reviewed the extracted data (P.G.F.), ensuring their completeness and accuracy. Any discrepancies were resolved through consensus between the two investigators, with a third investigator consulted if needed (R.M.L.). The data extraction sheet utilized for this process was based on the Cochrane form “Data collection form for intervention reviews for RCTs and non-RCTs—template,” ensuring standardized data collection across all studies.

The primary data sought from the articles included in this meta-analysis revolved around several key variables crucial for understanding head impacts in rugby players. Of utmost importance were the recorded head impact metrics, specifically PLA and PRA, as these parameters offer insights into the magnitude and directionality of impacts experienced by players during both practice and game sessions. In addition, information regarding the location of head impacts was crucial to delineate the anatomical regions most vulnerable to injury. Other sought-after data encompassed details about the wearable sensors employed, including brand, placement location on the body, and the kinematic components measured. Furthermore, data related to study characteristics such as the sport code, whether rugby union (RU) or rugby league (RL), participant demographics, study design, and outcome measures, were sought to ensure a comprehensive understanding of the research landscape pertaining to head impacts in rugby.

The main units of analysis, as presented by the authors of the included studies, were extracted and recorded in the data extraction sheet. These units of analysis were then unified to facilitate comparability across studies into mean and standard deviation [23]. Effect sizes and standard errors were calculated following procedures outlined in the Cochrane Handbook, ensuring accurate estimation for meta-analysis [24]. Data exclusively presented in graphical format were extracted through digitization using WebPlotDigitizer [25]. Overall, the data extraction process was conducted rigorously, facilitating data synthesis and analysis to address the research questions effectively.

### Risk of Bias and Publication Bias Assessment

Evaluating the risk of bias in prospective observational cohort studies is challenging due to the limited availability of validated and reliable assessment tools [26, 27]. However, the Newcastle–Ottawa Scale (NOS) is widely utilized as the most common tool for this purpose [26, 27]. The NOS quality assessment scale adapted for cross-sectional studies was used to evaluate the studies included in this review (Online Resource 2), The assessment relies on a star-based rating system, where each study can receive a maximum of nine stars, out of the following three main domains: selection of study group, comparability within sample, and ascertainment of the outcome [28, 29]. Quality evaluation was conducted independently by two authors, with any discrepancies resolved by a third author.

Selection models were used to assess publication bias in our meta-analysis. Classical methods, including Egger’s test and trim-and-fill, are commonly employed but have notable limitations, particularly in scenarios of heterogeneous effect sizes [30]. Selection models offer a more realistic perspective by assuming that publication bias favors statistically significant results and directly accommodating effect heterogeneity [30]. This decision underscores our commitment to robust reporting practices and the incorporation of methodologically sound approaches. Therefore, it was calculated using the Vevea and Hedges Weight-Function Model for Publication Bias [31].

### Statistical Analysis

The data were presented as mean values along with corresponding standard deviations. Calculations and analysis were conducted using the SPSS software (version 30.0.0.0), generating pooled means with 95% confidence intervals for collision dose among groups and subgroups with comparable data extracted from at least two similar studies. Information provided from different cohorts within the same study was considered as data derived from individual studies [32]. Meta-analysis was not performed when there were insufficient data for comparisons between studies within a particular group or subgroup. Studies were selected for inclusion if they reported data with their effect size, ensuring consistency across the analyses. The variables included in the meta-analysis were PLA, PRA, concussion PLA, and head-impact location. The *I*^2^ statistic, utilized to assess inconsistency and variation, was employed to evaluate heterogeneity, with values below 25% indicating minor, between 25 and 50% moderate, and above 75% substantial heterogeneity [33]. Given the considerable heterogeneity observed among studies, a random effects model was employed, utilizing the Hartung–Knapp–Sidik–Jonkman (HKSJ) method [34].

To systematically address heterogeneity in meta-analyses, we propose a two-step approach. First, analyze each potential moderator variable individually to assess its contribution to the total heterogeneity (using metrics such as the proportion of variance explained, *R*^2^; the variables assessed in this study included rugby code, acceleration threshold, brand and type of technology, year conducted, age of participants, level of play, and sex of participants). Second, combine the variables that individually account for the highest percentages of heterogeneity in a multivariable meta-regression model. Subgroup analyses will only be conducted if a substantial modifier of heterogeneity is detected, defined as a variable explaining at least 50% of the heterogeneity. This approach prioritizes variables with the most substantial influence and evaluates the combined explanatory power to identify the optimal model for reducing heterogeneity [35]. Such a standardized methodology ensures rigor, reproducibility, and transparency in the meta-regression process [36].

## Results

### Identification and Selection of Studies

**Fig. 1:**
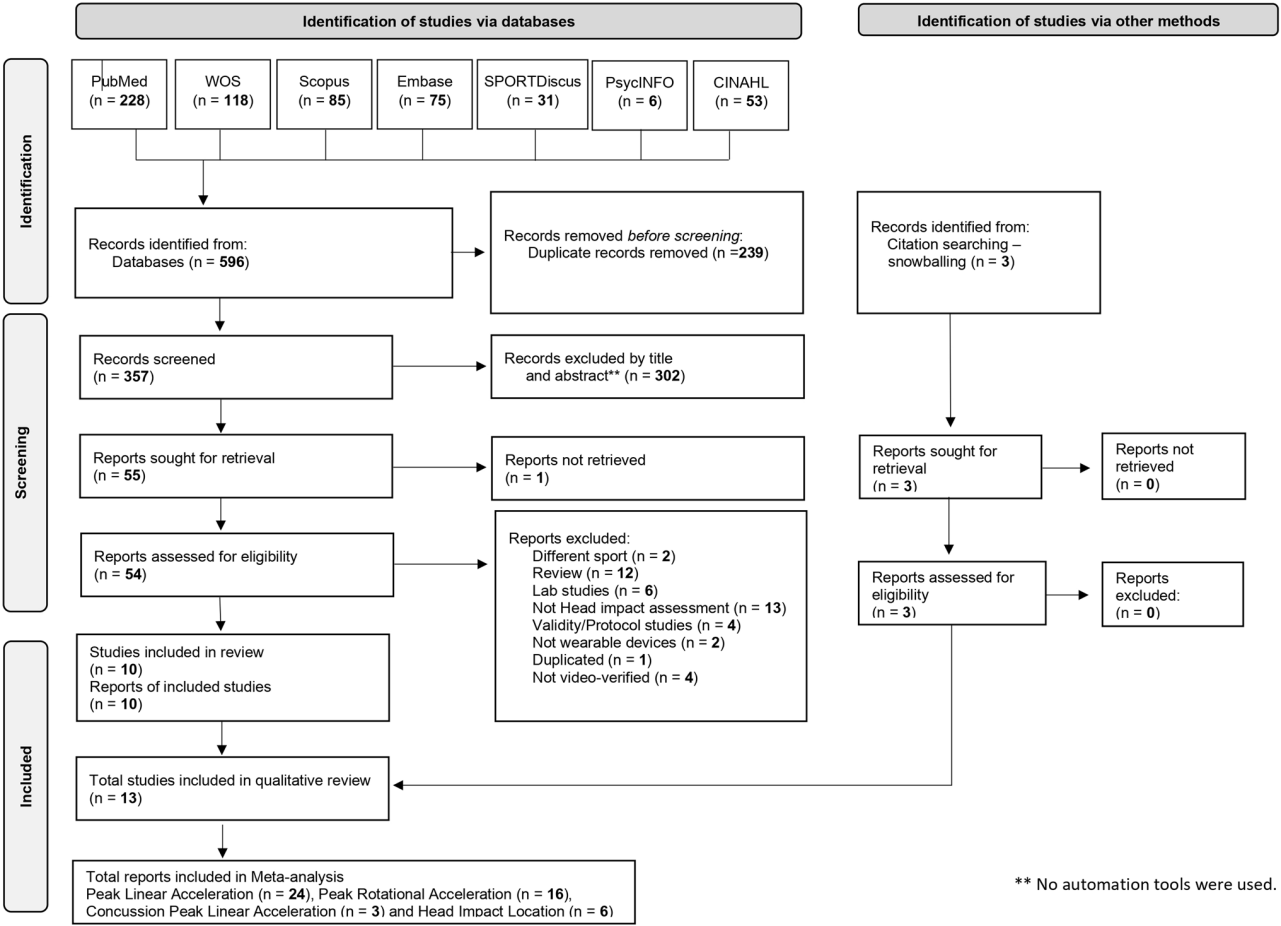
Flowchart showing the article selection process conducted by this review

A total of 596 studies were retrieved through the search strategy. These studies underwent the screening process (Fig. 1), resulting in 13 articles relevant to the research question that met the predefined inclusion criteria and were identified as suitable for further assessment.

### Study Characteristics

All 13 included studies are prospective cohort observational studies [37–49], collectively analyzing a total of 895 participants and 44,036 head impacts. All were conducted in Australasia and the Anglophone world, of which six studies (46.1%) were conducted in Oceania (Australia, New Zealand), four (30.7%) in Europe (UK), and the remaining three (23%) in North America (USA). Of the 13 studies identified, approximately 84% (11 out of 13) were published within the last 5 years, between 2020 and the present. This indicates a recent surge in research activity in the field of head impacts, potentially reflecting increasing awareness and interest in the topic. Furthermore, the fact that all of the articles were published within the last 10 years suggests a relatively recent focus on this area of study. The majority of the studies involved RU (77%), while the remaining focused on RL (23%). Within these studies athletes from junior (15.3%), college (30.7%), amateur (23%), and semi/professional (38.4%) levels were included. Overall, 61.5% of the studies only reported for matches, while the remaining 30.7% reported for trainings and matches. Individualized demographic details are summarized in Table 2.

**Table 2:** Included studies demographics

Study	Year	Country	Level	Code	Participants no. (sex)	Age years (mean ± SD)	Distribution no. (position)	Exposure (training/matches)	Reporting statistics				
									Mean (SD)	Median	IQR	95%	Other
Bussey et al. [37]	2023	New Zealand	Junior/amateur	RU	328 (M) (U13 = 60, U15 = 100, U19 = 78, S = 97)	12.5 ± 0.6 (U13), 14.8 ± 0.9 (U15), 16.9 ± 0.7 (U19), 22.5 ± 3.1 (S)	179 (Fwd), 156 (Bck)	(48/113)		✔	✔		✔
Bussey et al. [38]	2022	New Zealand	Amateur	RU	56 (M)	23.4 ± 3.4	30 (Fwd), 26 (Bck)	(–/5)		✔	✔	✔	✔
Carey et al. [39]	2019	Australia	Semi-professional	RL	8 (M)	25.5 ± 4.7	5 (Fwd), 3 (Bck)	(–/91)	✔				✔
Carey et al. [40]	2021	Australia	Junior	RL	21 (M)	15.5 ± 0.5	13 (Fwd), 8 (Bck)	–	✔	✔	✔		
Chan et al. [41]	2024	UK	Professional	RU	8 (M)	–	–	(–/1)	✔				✔
Field et al. [42]	2023	Australia	Professional	RU	10 (M)	25 ± 2	7 (Fwd), 3 (Bck)	(–/24)		✔	✔	✔	✔
Kieffer et al. [43]	2022	USA	College	RU	114 (F), 107 (M)	20.6 ± 1.3 (F) 20.6 ± 1.3 (M)	–	(17/26)		✔		✔	
King et al. [44]	2015	New Zealand	Amateur	RU	38 (M)	22 ± 4	–	(–/19)	✔				✔
Langevin et al. [45]	2021	USA	College	RU	23 (F)	20.17 ± 0.94	9 (Fwd), 5 (Bck), 9 (Adj)	(47/8)		✔	✔		
Manning et al. [46]	2020	USA	College	RU	130 (F)	19.95 ± 1.50) (season), 20.13 ± 1.43 (off-season)	–	(2/1)		✔	✔		✔
Tooby et al. [47]	2022	UK	Professional	RL	10 (M)	20.1 ± 3.3	8 (Fwd), 2 (Bck)	(–/31)		✔	✔	✔	
Waldron et al. [48]	2021	UK	Professional	RU	15 (M)	26 ± 4	9 (Fwd), 6 (Bck)	(–/10)	✔			✔	✔
Williams et al. [49]	2022	UK	College	RU	13 (F), 14 (M)	20.6 ± 2.1 (F), 20.7 ± 1.4 (M)	14 (Fwd), 13 (Bck)	(–/13)		✔	✔	✔	

Kinematics for head impacts were measured in studies by instrumented mouthguards (iMGs) (69.2%), intelligent headbands (15.3%), and skin patches (15.3%). Each included study reported PLA. Nevertheless, rotational forces were reported as PRA in nine studies (69.2%) and only as peak rotational velocity in three (23%), while the remaining study did not report on rotational forces (7.6%). Impact location on the head was reported in 46.1% of the studies. Regarding concussions, a total of 18 concussive impacts were collected in this review, 30.7% of the studies reporting kinematics for concussive impacts, and one providing incomplete data due to difficulties with collection [43–48]. Only 30.7% of the studies reported kinematics according to head impacts by player position (individual data for wearable devices and kinematics are presented in Tables 3 and 4, respectively).

**Table 3:** Wearables details reported individually

Study	Wearable	Brand	Body location	Kinematics included (output)	Components	Impact threshold considered for analysis	Total head impacts	RC	Incidence (head impacts × player-match/training hours)	Head impacts location (%)			
										Front	Side	Back	Top
Bussey et al. [37]	Instrumented mouthguard	Prevent Biometrics^®^	Upper dentition	PLA; PRA	3.2 kHz triaxial accelerometer and gyroscope	≥ 5 g	16450v	RU	U13: 3720 × 1000; U15: 6030 × 1000; U19: 8850 × 1000; premier: 10,650 × 1000	–	–	–	–
Bussey et al. [38]	Instrumented mouthguard	Prevent Biometrics^®^	Upper dentition	PLA; PRA; L	3.2 kHz triaxial accelerometer and gyroscope	≥ 5 g	825v	RU	2946 × 1000	30.54	24.72	22.54	22.18
Carey et al. [39]	Skin patch	X2Biosystems Inc	Over the mastoid process behind right ear	PLA; PRV; L	1.0 kHz triaxial accelerometer and gyroscope	≥ 20 g	536d	RL	8214 × 1000	35.4	36.2	21.3	5.3
Carey et al. [40]	Skin patch	X2Biosystems Inc	Over the mastoid process behind right ear	PLA; PRV; L	1.0 kHz triaxial accelerometer and gyroscope	≥ 20 g	564v	RL	7105 × 1000	12	79.2	7.2	1.4
Chan et al. [41]	Instrumented mouthguard	Protecht™	Upper dentition	PLA; PRA	9-axis IMU and 0.9 kHz triaxial accelerometer and gyroscope	≥ 10 g	146v	RU	13,727 × 1000	–	–	–	–
Field et al. [42]	Instrumented mouthguard	HitIQ ltd	Upper dentition	PLA; PRV; L	3.2 kHz triaxial accelerometer and gyroscope	≥ 10 g	655v	RU	6838 × 1000	30.53	51.90	9.61	7.93
Kieffer et al. [43]	Instrumented mouthguard	Prevent Biometrics^®^/Wake Forest	Upper dentition /upper palate	PLA; PRV	4.6 kHz triaxial accelerometer and gyroscope	≥ 5 g	1084v	RU	Females: 2123 × 1000; male: 6351 × 1000		–	–	–
King et al. [44]	Instrumented mouthguard	X2Biosystems Inc	Upper dentition	PLA; PRV; L	1.0 kHz triaxial accelerometer and gyroscope	≥ 10 g	20687v	RU	54,586 × 1000	28.9	44.6	23.8	2.6
Langevin et al. [45]	Instrumented headband	Triax Technologies, Inc	Headband	PLA; PRV; L	900 MHz triaxial accelerometer and gyroscope	≥ 15 g	120v	RU	11,356 × 1000	39	28	28	5
Manning et al. [46]	Instrumented headband	Artaflex, Inc	Occipital bone	PLA; PRA	3.0 kHz triaxial accelerometer and gyroscope	≥ 15 g	151v	RU	Practice: 3833 × 1000; game: 1805 × 1000	–	–	–	–
Tooby et al. [47]	Instrumented mouthguard	Prevent Biometrics^®^	Upper dentition	PLA; PRA	3.2 kHz triaxial accelerometer and gyroscope	≥ 5 g	1622v	RL	39,341 × 1000	–	–	–	–
Waldron et al. [48]	Instrumented mouthguard	Protecht™	Upper dentition	PLA; PRA	9-axis IMU and 0.9 kHz triaxial accelerometer and gyroscope	≥ 10 g	978v	RU	4902 × 1000	–	–	–	–
Williams et al. [49]	Instrumented mouthguard	Protecht™	Upper dentition	PLA; PRA	9-axis IMU and 0.9 kHz triaxial accelerometer and gyroscope	≥ 10 g	218v	RU	Females: 4220 × 1000; males: 7786 × 1000	–	–	–	–

*d* direct to head verified impacts, *IMU* inertial magnetic unit, *kHz* kilohertz, *L* localization of impact, *MHz* megahertz, *PLA* peak linear acceleration, *PRA* peak rotational acceleration, *PRV* peak rotational velocity, *RC* rugby code, *RL* rugby league, *RU* rugby union, *v* verified impacts

**Table 4:** Studies’ individual reports on kinematics

Study	RC	No injury (Mean ± SD)				No. concussions	Injury (mean ± SD)
		PLA (*g*)		PRA (rad/s^2^)			PLA (*g*)
		Training	Game	Training	Game		
Bussey et al. [37]	RU	12.9 ± 2.3 (U13) 12.05 ± 2.35 (U15) 12.6 ± 2.7 (U19) 12.1 ± 2.8 (S)	11.81 ± 2.38 (U13) 13.42 ± 3.73 (U15) 13.36 ± 3.59 (U19) 13.4 ± 6.15 (S)	1626.29 ± 849.3 (U13) 1534.99 ± 984.06 (U15) 1181.57 ± 1025.93 (U19) 1119.98 ± 836.59 (S)	1334.97 ± 585.12 (U13) 1167.31 ± 799.89 (U15) 975.63 ± 945.25 (U19) 849.22 ± 734.46 (S)	N/R	–
Bussey et al. [38]	RU	–	17.1 ± 9.4	–	1329.1 ± 684.6	N/R	–
Carey et al. [39]	RL	–	34.2 ± 18.0	–	–	6	76 ± 16.9
Carey et al. [40]	RL	–	34.1 ± 16.1	–	–	–	–
Chan et al. [41]		–	18.8 ± 14.3	–	1592.9 ± 1368.8	–	–
Field et al. [42]	RU	–	23.05 ± 5.26	–	1835.62 ± 461.17	–	–
Kieffer et al. [43]	RU	–	15.3 ± 28 (F), 14.6 ± 22.3 (M)	–	–	6	64.1 ± 30.2
King et al. [44]	RU	–	22.2 ± 16.2	–	3902.9 ± 3948.8	2	74.8 ± 28.2
Langevin et al. [45]	RU	29.9 ± 2.1	31.9 ± 2.6	–	–	4	39.8 ± 20.7
Manning et al. [46]	RU	25.9 ± 3.4	22.9 ± 2.4	1445.6 ± 409.21	1309.18 ± 276.92	–	–
Tooby et al. [47]	RL	–	7.3 ± 1.3	–	625 ± 144.3	–	–
Waldron et al. [48]	RU	–	16.80 ± 2.79	–	1224.09 ± 436.15	–	–
Williams et al. [49]	RU	–	11.6 ± 6.8 (F), 12.5 ± 6 (M)	–	800.2 ± 524.4 (F), 849.4 ± 432.3 (M)	–	–

*F* female, *M* male, *N/R* not reported, *PLA* peak linear acceleration, *PRA* peak rotational acceleration, *RC* rugby code, *RL* rugby league, *RU* rugby union, *S* senior, *SD* standard deviation, *U13* under 13, *U15* under 15; *U19* under 19

### Quality Assessment

The methodological quality of the included studies was fair to good; scores ranged from 4 to 8 on the total 9-item scale (Table 5). The inclusion criteria and the nature of this study already screened for a certain baseline quality. Considering that the ascertainment of head impacts is measured by validated instruments in all the included studies, and data collection is automatically processed and even video verified in some of the cases, these directly resulted in a “fair” quality baseline in NOS. Items that were rarely scored were related to sample selection.

**Table 5:** Studies’ resultant scores on the Newcastle–Ottawa scale

Study	Selection (5 max)	Comparability (1 max.)	Exposure (3 max.)	Total (interpretation)
Bussey et al. [37]	✔	✔	✔✔✔	7/9 (“Good”)
Bussey et al. [38]	✔✔✔	✔	✔✔✔	7/9 (“Good”)
Carey et al. [39]	✔✔	✔	✔✔	5/9 (“Fair”)
Carey et al. [40]	✔✔✔✔		✔✔	6/9 (“Fair”)
Chan et al. [41]	✔✔		✔✔	4/9 (“Fair”)
Field et al. [42]	✔✔	✔	✔✔✔	6/9 (“Fair”)
Kieffer et al. [43]	✔✔✔	✔	✔✔	6/9 (“Fair”)
King et al. [44]	✔✔	✔	✔✔✔	6/9 (“Fair”)
Langevin et al. [45]	✔✔		✔✔	4/9 (“Fair”)
Manning et al. [46]	✔✔✔✔	✔	✔✔✔	8/9 (“Good”)
Tooby et al. [47]	✔✔	✔	✔✔✔	6/9 (“Fair”)
Waldron et al. [48]	✔✔	✔	✔✔✔	6/9 (“Fair”)
Williams et al. [49]	✔✔	✔	✔✔✔	6/9 (“Fair”)

### Head Impacts—Peak Linear Acceleration

PLA emerged as the most frequently reported variable across all included studies, with each study providing data on this metric. Despite the consistency in reporting, a substantial degree of heterogeneity was observed in the pooled analysis, as indicated by the *I*^2^ statistic. To address this variability, we conducted a meta-regression analysis to identify the primary factor contributing to the observed heterogeneity within our dataset.

Our analysis revealed that the wearable brand and the recording threshold reported in the included studies were significant determinants of variability, each explaining a substantial 82% of the observed heterogeneity (*R*^2^ = 0.82). The “type of sensor” emerged as the second most significant moderator, accounting for 76.3% (*R*^2^ = 0.76), followed by the year of the study resulting in 67% (*R*^2^ = 0.67). When combining these variables in a multivariate model, the predictors “year” and “brand” together accounted for 91.8% (*R*^2^ = 91.8) of the heterogeneity, with residual heterogeneity estimated at *τ*^2^ = 5.01.

**Fig. 2:**
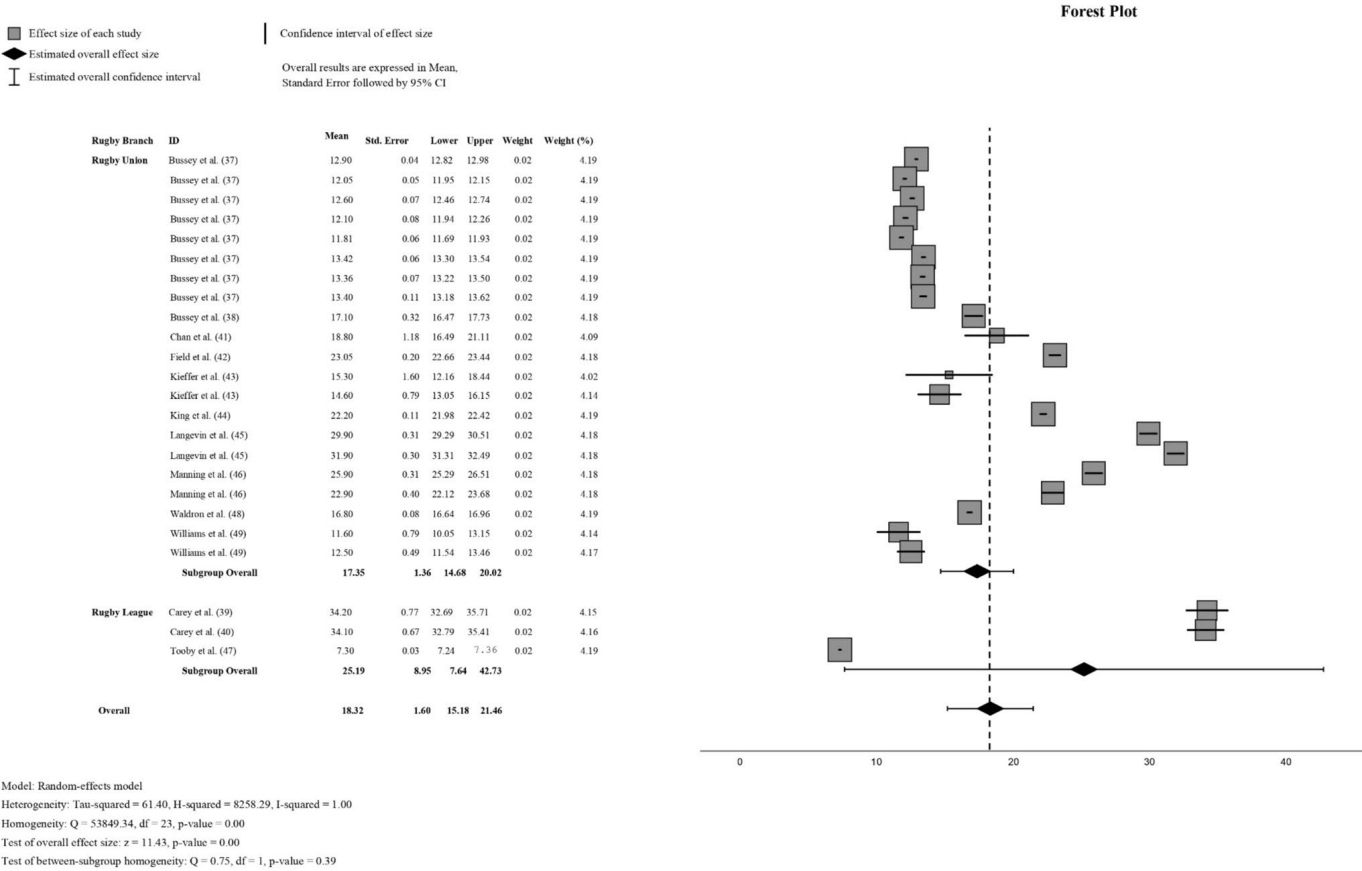
Peak linear acceleration of rugby head impact meta-analysis, subgrouped by rugby union and rugby league

To complement this, the subgroup analysis stratified the dataset by rugby code (RU versus RL) owing to its practical relevance, despite explaining only 7% of the overall heterogeneity. This approach provided a more detailed examination of how these moderating variables influenced the pooled PLA results. For RU, the pooled PLA was 17.35 *g* (95% CI 14.68–20.02 *g*), while for RL, it was 25.19 *g* (95% CI 7.64–42.73 *g*). When combined, the pooled PLA across both rugby codes was 18.32 *g* (95% CI 15.18–21.46 *g*). The forest plot and extended results are presented in Fig. 2. A supplementary document (Online Resource 3) is available to facilitate the interpretation of the forest plot.

The subgroup analysis revealed distinct PLA estimates for different recording thresholds: for a threshold of 5 *g*, the meta-analysis showed a PLA of 12.91 *g* (95% CI 11.58–14.24 *g*); whereas for a threshold of 20 *g* or more, the PLA was 34.14 *g* (95% CI 33.15–35.13 *g*). Regarding brands, Prevent Biometrics® reported the lowest pooled PLA, with an estimate of 12.91 *g* (95% CI 11.58–14.24 *g*). In contrast, the Triax Technologies, Inc. subgroup showed significantly higher overall results, with a pooled PLA of 30.90 *g* (95% CI 28.98–32.82 *g*). A detailed summary of these subgroup analyses is presented in Table 6.

**Table 6:** Pooled PLA results and heterogeneity metrics by subgroups

Subgroup	*N* (reports)	Pooled PLA (*g*)	95% CI (*g*)	*R*^2^	*τ*^2^
Prevent Biometrics®	12	12.91	11.58–14.24	91%	5.53
Protecht™	4	14.87	11.56–18.18	80.8%	11.82
HitIQ ltd	1	23.05	22.66–23.44	Only one report	
Artaflex, Inc	2	24.41	21.51–27.31	92.7%	4.50
X2Biosystems Inc	3	30.14	22.32–37.95	22.8%	47.60
Triax Technologies, Inc	2	30.90	28.98–32.82	96.7%	2.00
Threshold > 5 g	12	12.91	11.58–14.24	91%	5.53
Threshold > 10 g	6	17.51	13.67–21.35	62.7%	22.97
Threshold > 15 g	4	27.66	23.71–31.60	73.6%	16.25
Threshold > 20 g	2	34.14	33.15–35.13	99.9%	0.00

*CI* confidence interval, *PLA* peak linear acceleration, *R*^*2*^ coefficient of determination, *τ*^*2*^ Tau-squared

### Head Impacts—Peak Rotational Acceleration

**Fig. 3:**
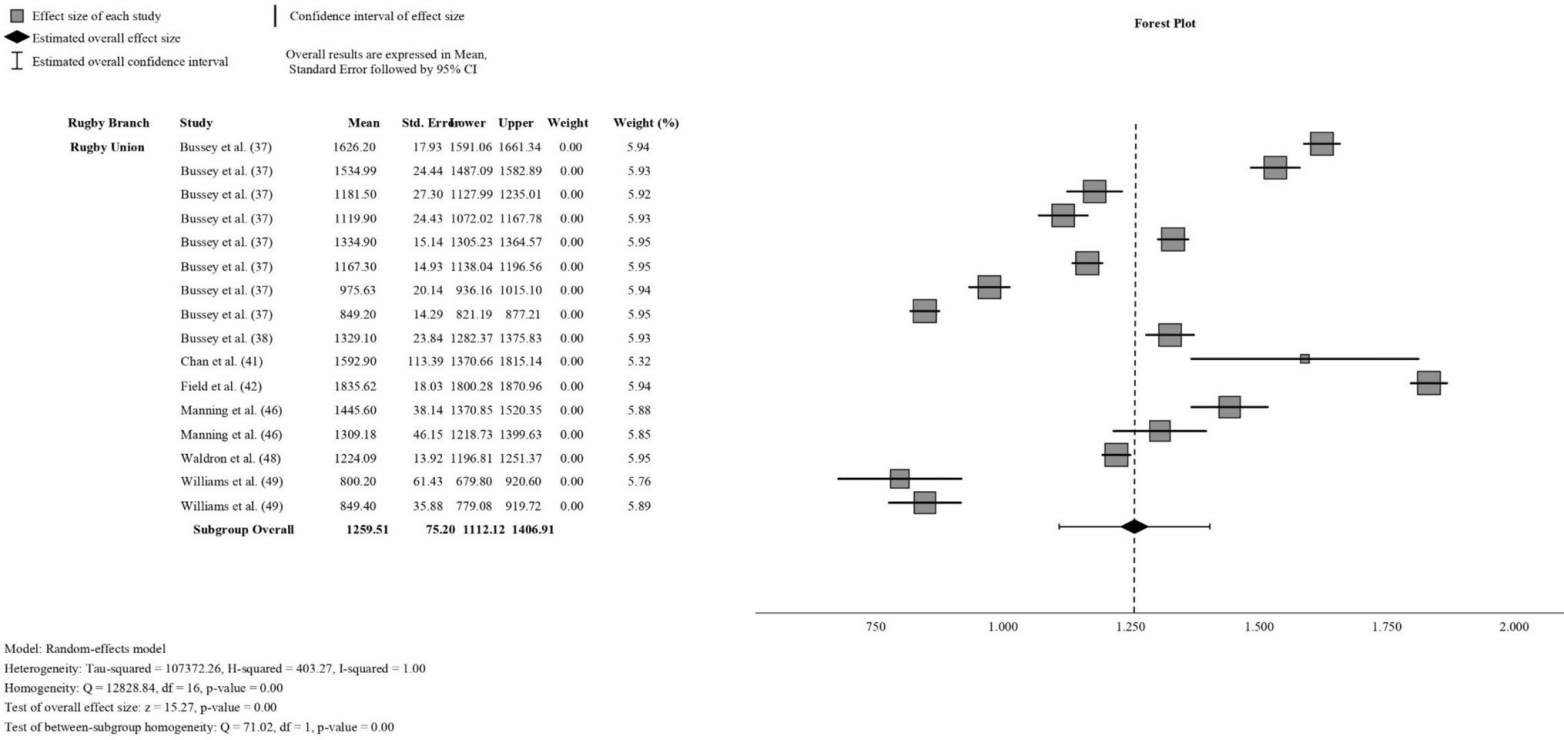
Peak rotational acceleration of rugby head-impact meta-analysis in rugby union reports

Regarding rotational forces, PRA was a key variable analyzed in this study. Despite the inclusion of various studies, substantial heterogeneity was observed with an *I*^2^ = 0.99 in the pooled analysis. A meta-regression analysis was conducted, focusing on understanding the factors contributing to the observed high level of heterogeneity. The analysis revealed that the brand of the inertial sensor used in the studies was the most influential variable, explaining 20% of the observed heterogeneity (*R*^2^ = 0.201), followed by the level of play, which accounted for 14% (*R*^2^ = 0.141). Upon further investigation, a meta-regression model combining these variables explained only 45% of the heterogeneity, which was deemed insufficient to justify subgrouping by these variables. Subsequently, a practical subgrouping by rugby code was performed, yielding a combined PRA of 1221.89 rad/s^2^ (95% CI 1065.08–1378.70 rad/s^2^). However, as the RL subgroup contained only one report (Tooby et al. [47]; PRA of 625 rad/s^2^; 95% CI 617.98–632.02 rad/s^2^), it was excluded from the forest plot, focusing instead on RU results. Despite the observed heterogeneity, the meta-analysis produced a pooled PRA estimate of 1259.51 rad/s^2^ (95% CI 1112.12–1406.91 rad/s^2^) for RU. The forest plot and extended results are shown in Fig. 3.

### Impact Location on Head

Head impact location was another critical variable analyzed in this study. A proportion meta-analysis was conducted using the raw distribution reported in six studies to determine the prevalence of head impacts across different locations.

Meta-analysis with practical subgrouping by rugby code revealed that side impacts accounted for the majority of head impacts, comprising 38% of all reported incidents for RU (proportion = 0.38, 95% CI 0.25–0.50) and 58% for RL (proportion = 0.58, 95% CI 0.16–1.00). Frontal impacts were also prevalent, constituting 31% of head impacts in RU (proportion = 0.31, 95% CI 0.27–0.35) and 24% in RL (proportion = 0.24, 95% CI 0.01–0.46). Back impacts were less frequent, representing 21% of all reported head impacts in RU (proportion = 0.21, 95% CI 0.13–0.28) and 15% in RL (proportion = 0.15, 95% CI 0.00–0.30), while impacts on the top of the head remained the least common, comprising only 9% of reported incidents in RU (proportion = 0.09, 95% CI 0.01–0.18) and 4% in RL (proportion = 0.04, 95% CI 0.01–0.08).

**Fig. 4:**
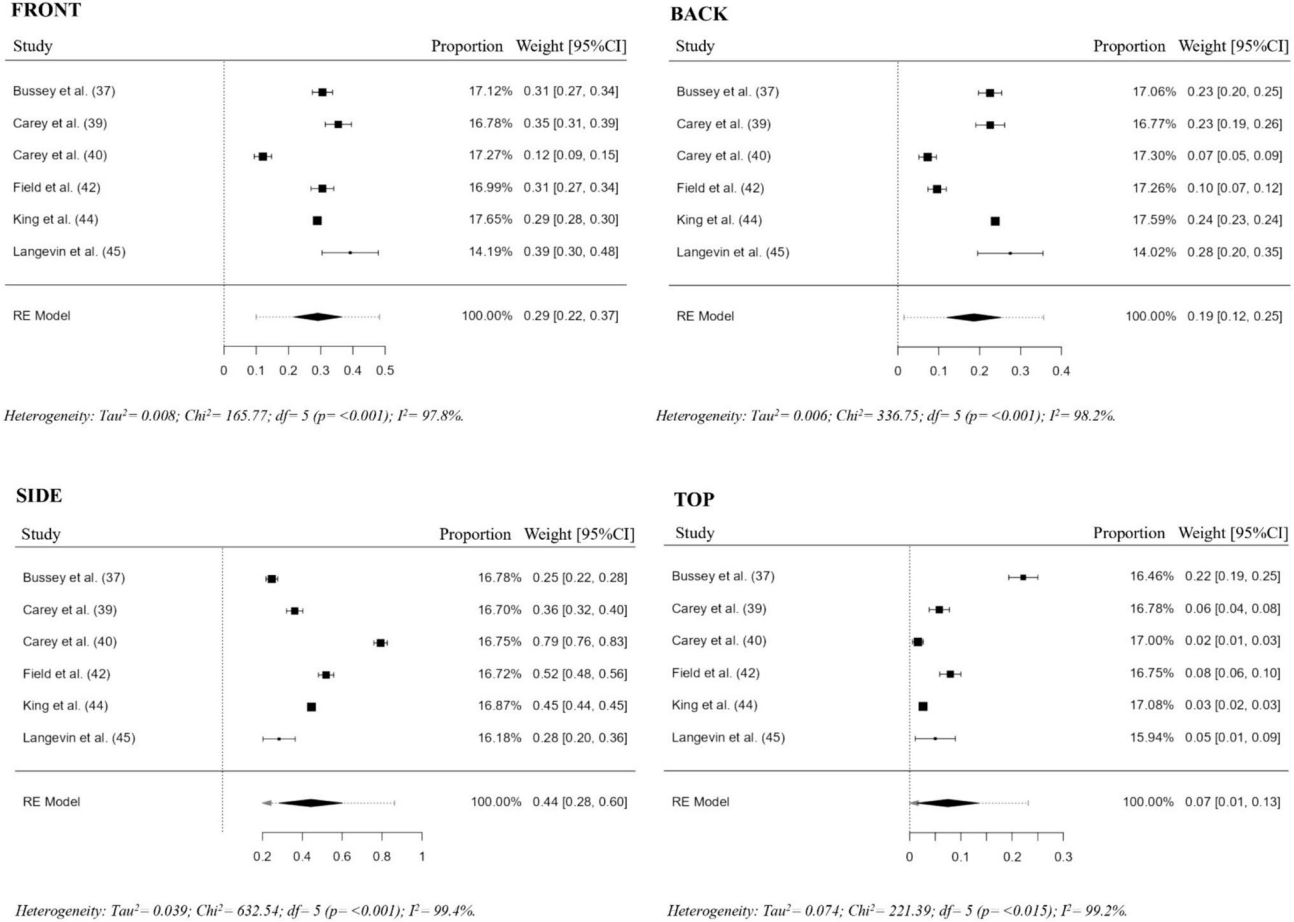
Head-impact location proportion meta-analysis (front, side, back, and top)

To provide a unified perspective on rugby as a contact sport, a pooled analysis was conducted to combine data from both codes. This approach emphasizes the overall prevalence of head impacts in rugby, underscoring its inherent risk profile while allowing for detailed discussions about code-specific variations. Results are visually presented in the forest plot and extended in Fig. 4.

### Concussive Impacts

The analysis of concussive events constituted a significant aspect of this study, with data extracted from four studies focusing on the kinematics of these events. Pooling the data from a total of 18 concussive events, a meta-analysis was conducted with moderate heterogeneity observed in the pooled analysis, with an *I*^2^ value of 60%.

**Fig. 5:**
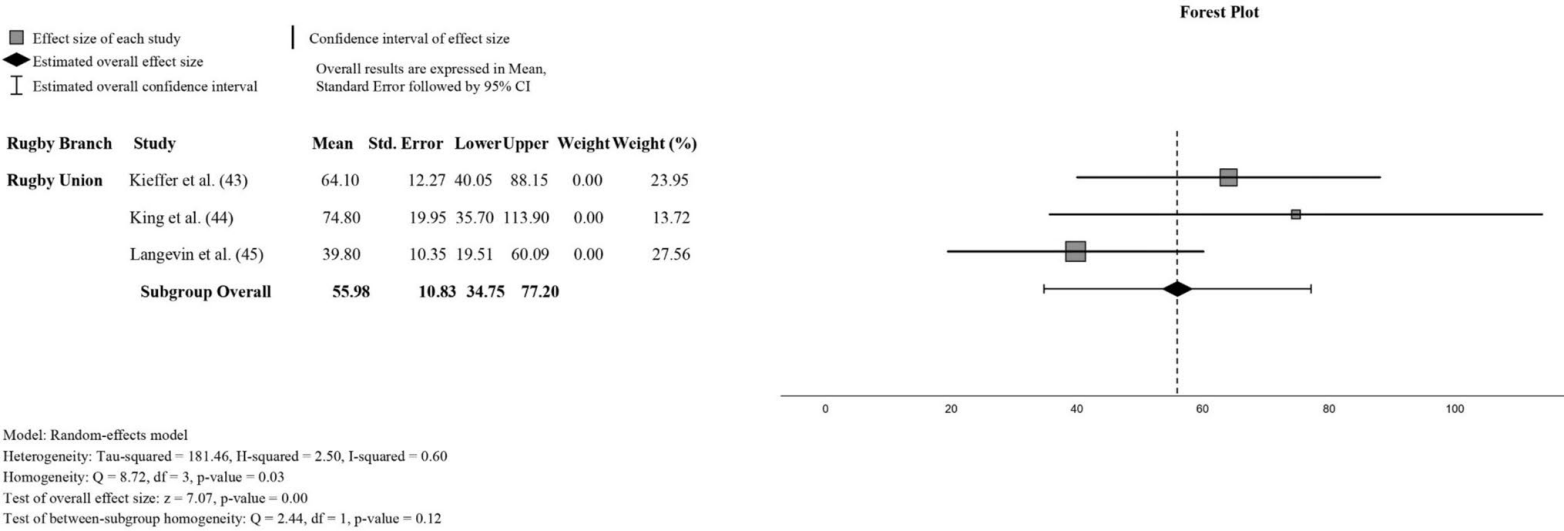
Concussive impacts meta-analysis of rugby union reports

The subgroup analysis revealed distinct PLA estimates for different rugby codes. RU cohorts exhibited a PLA of 55.98 *g* (95% CI 34.75–77.20 *g*), while the RL subgroup, represented by only one cohort, reported a PLA of 76 *g* (95% CI 62.52–89.48 *g*). The combined pooled data showed a PLA of 63.01 *g* (95% CI 45.53–80.49 *g*). The forest plot presents results exclusively for RU owing to the exclusion of the RL cohort, with extended results presented in Fig. 5.

## Discussion

This study represents a seminal systematic review, incorporating meta-analyses, to measure both the intensity and sites of head impacts endured by rugby athletes utilizing wearable technology. Unlike previous reviews by King et al. [10], which encompassed laboratory investigations, as well as studies by Nguyen et al. [7] and Brennan et al. [8], which encompassed a variety of sports, this systematic review exclusively concentrated on real-world impacts occurring specifically within rugby.

The results suggested no evidence of publication bias across the included analyses. Specifically, the Vevea and Hedges Weight-Function Model for Publication Bias reported a *p*-value of 0.41 for PLA, indicating no significant bias. Similarly, for PRA, the *p* value was 0.98, indicating no substantial evidence of bias. In addition, in concussive events analysis, the *p* value was 0.98, further supporting the absence of publication bias.

The observed heterogeneity in our meta-analysis underscores the complexity inherent in quantifying the magnitude and location of head impacts across various studies in rugby. Factors contributing to this heterogeneity include differences in study populations and methodological approaches utilized across the included studies. While our meta-analysis aimed to provide a comprehensive overview of head impact characteristics in rugby, the substantial heterogeneity observed highlights the need for cautious interpretation of the aggregated results.

The subgroup analysis highlighted notable differences in PLA between RL and RU, reflecting the distinct gameplay dynamics of each code. RL reported a higher pooled PLA of 25.19 *g* (95% CI 7.64–42.73 *g*) compared with 17.35 *g* (95% CI 14.68–20.02 *g*) for RU. This discrepancy may stem from the distinct gameplay demands, as tackles and player interactions differ significantly, driven by the structural and tactical distinctions between these rugby codes [50, 51]. In RL, a deeper defensive line results in higher-speed collisions, concentrating impacts among forwards who frequently engage in high-momentum tackles [50]. In contrast, RU’s prolonged phases of play, featuring rucks, mauls, and open-field running, distribute impacts across a wider range of intensities [51]. These differences reflect how gameplay structure influences head-impact severity in each rugby code.

The variability in PLA for RL (95% CI 7.64–42.73 *g*) compared with RU (95% CI 14.68–20.02 *g*) also underscores the impact of limited data in RL, with fewer cohorts contributing to the analysis. This variability highlights the need for further studies to enhance the precision of estimates for RL and to better understand its specific head impact profile.

This study revealed distinct trends in head impact metrics across different recording thresholds. For instance, at a 5 *g* acceleration threshold, the estimated mean incidence of head impacts was 12.91 *g* (95% CI 11.58–14.24 *g*), whereas, at a 20 *g* force threshold, the mean incidence increased to 34.14 *g* (95% CI 33.15–35.13 *g*). These findings align with previous research highlighting the threshold dependence of head impact metrics, where higher thresholds tend to underestimate incidence, while lower thresholds may underestimate impact severity [10]. These findings provide valuable insights into the impact of different recording thresholds on PLA measurements and underscore the importance of considering this factor in future studies and clinical practice. A recent study underscores the need to be cautious about this underestimation of head impact exposure, more specifically referring to iMGs research [52].

When analyzing the subgrouping results in Table 6, it is evident that the brand aligns closely with the sensor type. However, it is logical that brand plays a more decisive role in explaining heterogeneity, as differences within the type of sensor can often be attributed to variations between brands. This highlights the importance of examining the feasibility and characteristics of the included sensor brands, as these factors can significantly influence the outcomes and interpretations of the data.

Studies reported for Prevent Biometrics iMGs an on-field positive predictive value (PPV) for the boil and bite model of 81.6% (95% CI 75.5–87.8%) [5] and 89% (95% CI 87.0–92.0%) for the custom fit [53]. Conversely, the X2Biosystems skin patch yielded an on-field PPV of 16.3% (95% CI 15.5–17.0%) [5], while the HitIQ iMGs showed 92.4% [42]. Studies for Protecht iMGs have not addressed true/false positive impacts. However, this technology seems valid for on-field use when verified by video [54]. Furthermore, strong agreement was observed in laboratory tests (concordance correlation coefficient = 0.90) and on-field assessments (concordance correlation coefficient = 0.97) when compared with an anthropomorphic test device (ATD) [54, 55]. Regarding Artaflex technology, in a laboratory-based study, which compared it with ATD, it accurately predicted PLA and PRA if a correction algorithm was used [56]. Otherwise, it tended to overestimate PLA at the center of mass of the head by an average of 47% and the error in the PRA determined by the algorithm was less than 10% [56]. While the brand of the inertial sensor plays a substantial role, considering advancements in sensor technology over time in conjunction with the brand provides an even more comprehensive understanding of our results. As sensors become more integrated, comfortable, and user-friendly, there has been a growing need for a smooth monitoring experience in both sport and clinical settings [57]. These findings provide valuable insights into the variation in rotational acceleration measurements across different sensor brands and underscore the importance of considering sensor characteristics in future research and clinical practice. Moreover, it is important to highlight that there is currently no standardized signal processing approach for the various iMGs. This lack of standardization can lead to variability in kinematic results, particularly during field use, as some systems may produce significantly higher head kinematic values than others [53]. The ongoing rapid advancements in hardware, firmware, and data-processing algorithms to improve the validity of kinematic data pose additional challenges for researchers and practitioners. Frequent updates to proprietary algorithms, often lacking independent validation, further complicate the comparability and consistency of results. A potential approach to mitigate these challenges is suggested by Tierney et al., who propose standardized frameworks for algorithm validation and enhanced transparency in data processing methodologies [58].

Furthermore, our analysis underscores the importance of considering clinical relevance and context when interpreting head impact data. Understanding which aspects of head impacts are of greatest interest, such as the total number of impacts or the severity of higher impacts, is crucial for informing study design and subsequent clinical decisions. Recent publications have highlighted, through animal models, the cumulative effects of repeated head impacts at intensities insufficient to cause concussion. These studies demonstrated alterations in the blood–brain barrier, changes in neuronal metabolism, neuroinflammation, and deposits of tau protein [59]. A recently published review also observed anatomical changes in amateur athletes following repeated head impacts at sub-concussive intensities [60]. These structural changes appear to be more closely associated with the cumulative effect of impacts rather than a single event, underscoring the importance of incorporating the cumulative incidence of impacts into study designs.

Moreover, the recommendation by King et al. to use median instead of mean statistics for a more accurate representation of central tendency [10] deserves attention. The predominance of median statistics (9 out of 13 studies) in our meta-analysis suggests that the pooled data are robust and suitable for further analysis, despite the heterogeneity introduced by varying recording thresholds.

In comparing the combined two rugby code PLA values, our study yielded a pooled estimate of 18.32 *g* (95% CI 15.18–21.46 *g*), which is lower than the values reported in two other studies [7, 61]. Exploring our findings suggests that rugby impacts involve lower linear forces compared with both American Football 25.43 *g* (95% CI 19.18–31.69 *g*) [7] and mixed sports 22.89 *g* (95% CI 20.69–25.08 *g*) [61]. The focus on a single sport provides a structured framework for assessing impact magnitudes, yet the inherent variability within our dataset (emerging from differences in sensors), variations between RU and RL, and the inclusion of distinct cohorts, emphasizes the need for cautious interpretation. Nevertheless, by analyzing these differences, we can better understand the variations in impact forces across different sports. Despite variations in concussion frequency reported across different levels of play in rugby [62], it remains unclear whether biomechanical variables, such as PLA, differ significantly across these levels. Given the increased intensity and physical demands observed in higher-level play [62], we conducted a post hoc statistical analysis to assess whether PLA varied significantly across levels of play. The analysis revealed no significant differences (*p* = 0.575), supporting the observation that head impact magnitudes are relatively consistent across subgroups. In addition, when assessed as a modifying variable, PLA accounted for no more than 15% of the heterogeneity, reinforcing its limited role in explaining variation. Contrary to expectations, these findings suggest the magnitude of head impacts does not vary considerably between different levels of play. Further studies are warranted to confirm these findings and explore potential factors contributing to head-impact severity across different levels of play. This is consistent with the findings of Broglio et al., who observed similar results when assessing head impact severity across various levels of American Football competition [63].

Examining non-concussive PRA in the RU code, our study yielded a pooled estimate of 1259.51 rad/s^2^ (95% CI 1112.12–1406.91 rad/s^2^). Interestingly, this estimate is comparable to that reported in the study on mixed sports (1290.13 rad/s^2^, 95% CI 1050.71–1529.55 rad/s^2^) [61], suggesting that rugby impacts may involve similar or slightly less intense rotational forces than those experienced in a mixed sports setting. However, our PRA estimate in rugby remains lower than the reported estimate for game-related American Football head impacts [7]. Moreover, the resulting differences might be due to discrepancies in assessing impacts within the included sensors and their development through the years.

Transitioning to concussive impacts, despite excluding the RL cohort from the forest plot, the pooled data were included to offer a broader perspective on rugby as a whole. This approach provides a unified framework for comparing rugby impacts with those of other contact sports, many of which aggregate data across disciplines. By integrating both pooled and code-specific estimates, the analysis highlights rugby’s unique impact dynamics while facilitating meaningful cross-sport comparisons. It is worth noting that the RL cohort consisted of female players, while RU cohorts were male, reflecting both rugby code and sex-based subgrouping.

The subgroup analysis revealed distinct PLA estimates for different rugby codes. RU cohorts exhibited a PLA of 55.98 *g* (95% CI 34.75–77.20 *g*), while the RL subgroup, represented by only one cohort, reported a PLA of 76 *g* (95% CI 62.52–89.48 *g*). The combined pooled data showed a PLA of 63.01 *g* (95% CI 45.53–80.49 *g*). This outcome contrasts with previous observations in male participants from mixed sports backgrounds, where higher PLA estimates were documented. For instance, Brennan et al. [8] documented a PLA of 98.68 *g* (95% CI 82.36–115.00 *g*), while Sundaram et al. [61] reported a PLA of 85.56 *g* (95% CI 69.34–101.79 *g*) within similar male mixed sports cohorts. This implies potential disparities in the characteristics and intensity of concussive impacts between rugby-specific and mixed sports settings. While the exact reasons for these discrepancies remain speculative, they may stem from variations in playing style, rule enforcement, or protective equipment usage across different sports. Unfortunately, data specific to concussive events in rugby were insufficient for rotational acceleration comparison. These findings underscore the importance of considering sex-based and rugby code differences in head impact kinematics and their potential implications for concussion risk and management.

Overall, while our findings indicate some similarities in impact magnitudes across sports, particularly in linear acceleration, significant variations exist, especially in the context of concussive events. It is crucial to acknowledge that our results, while suggestive, do not provide a comprehensive understanding owing to the relatively low number of concussions assessed. Therefore, further research with larger sample sizes and comprehensive concussion data is imperative to draw more robust conclusions regarding the comparative impact magnitudes across different sports.

This meta-analysis provides valuable insights into the distribution of head impacts in rugby, shedding light on the location and frequency of these incidents. Interestingly, our findings reveal distinct patterns in head impact location within the rugby context, differentiating between its own codes. This probably stems from the aforementioned distinctions in gameplay dynamics, with RL’s structured tackles emphasizing lateral impacts and RU’s rucks and mauls distributing impacts more broadly [51].

The combined rugby code pooled data on head-impact locations provide a broader perspective on the sport’s overall risk profile. Comparing this with a similar contact sport, such as American Football, is valuable for understanding the shared biomechanical challenges inherent in high-impact sports. Our analysis revealed that the distribution of head impact locations in rugby is remarkably similar to that observed in American Football, despite differences in equipment and gameplay techniques. Side impacts emerged as the most common in both sports, followed by frontal and back impacts, while impacts on the top of the head remained the least frequent. These slight variations in proportions are 44% versus 45% for side impacts, 29% versus 26% for frontal impacts, 19% versus 20% for back impacts, and 7% versus 5% for top impacts. This underscores the shared biomechanical challenges inherent in high-impact sports. Such similarities highlight the importance of cross-sport comparisons in understanding head impact dynamics and developing broader injury prevention strategies that account for both gameplay and equipment differences.

Nevertheless, comparing these findings with the available literature on American Football, particularly a study focusing on youth athletes, reveals notable differences in the distribution of head impacts [64]. While the front of the helmet is reported in the literature as the most common impact location, accounting for 31–52% of total head impacts, our findings do not align with this observation. However, the literature agrees that, in American Football, impacts on the top of the helmet are typically the least common, stating a range of 5.5–18% of head impacts [64]. Further research may be warranted to explore the factors contributing to variations in impact location and their potential implications for player safety and performance.

It is important to highlight that our study represents the first attempt to pool and analyze data specifically on head impact location in rugby using wearable technology. Understanding the distribution of head impacts across different locations is clinically significant for several reasons. Firstly, it provides valuable insights into injury prevention strategies tailored to the specific demands of rugby. Secondly, it enhances our understanding of the biomechanics of head impacts in the sport, potentially informing the development of improved protective equipment. Lastly, it underscores the importance of future research efforts to explore head impact location in various contact sports comprehensively.

### Limitations

The main limitation of our study is the heterogeneity present in the existing studies. The variability in the technologies used to measure head impacts across studies introduces inconsistency, and challenges comparability. Despite our thorough search, the scarcity of relevant literature limits the breadth and depth of our analysis. The field of wearable head-impact assessment, particularly in rugby players, is relatively novel, leading to a lack of standardized protocols and methodologies. The absence of established guidelines may have contributed to inconsistencies in data reporting. Rugby head impacts involve dynamic and multifaceted forces that are difficult to standardize, influenced by player variables and environmental factors.

These limitations highlight the need for future research to address methodological inconsistencies, expand the available literature, and enhance standardization in head impact assessment protocols.

## Conclusion

In summary, this meta-analysis provides valuable insights into the biomechanics of head impacts in rugby, leveraging wearable sensor technology. Findings indicate significant variability in PLA and PRA, with PLA averaging 17.35 *g* for RU and 25.19 *g* for RL, and PRA reaching 1633.48 rad/s^2^ in RU cohorts. Impact location analysis reveals side impacts as the most prevalent (44%), followed by frontal (29%), back (19%), and top impacts (7%). Despite these findings, the study’s interpretation is constrained by methodological heterogeneity across studies. Future research should prioritize standardized protocols and cumulative impact analysis to advance head injury prevention strategies in rugby.

## Supplementary Information

Below is the link to the electronic supplementary material.

Supplementary file1 (DOCX 27 KB)

Supplementary file2 (DOCX 22 KB)

Supplementary file3 (DOCX 41 KB)

## Declarations

### Data availability

The datasets used and/or analyzed during the current review and meta-analysis are available from the corresponding author upon reasonable request.

## References

[1] Hausler J, Halaki M, Orr R. Application of global positioning system and microsensor technology in competitive rugby league match-play: a systematic review and meta-analysis. Sports Med. 2016;46(4):559–88. 10.1007/S40279-015-0440-6/TABLES/6. 26714810 10.1007/s40279-015-0440-6

[2] West SW, Shill IJ, Bailey S, et al. Injury rates, mechanisms, risk factors and prevention strategies in youth rugby union: what’s all the ruck-us about? A systematic review and meta-analysis. Sports Med. 2023;53(7):1375–93. 10.1007/S40279-023-01826-Z/TABLES/4. 37191819 10.1007/s40279-023-01826-z PMC10290028

[3] Patricios JS, Schneider KJ, Dvorak J, et al. Consensus statement on concussion in sport: the 6th International Conference on Concussion in Sport–Amsterdam, October 2022. Br J Sports Med. 2023;57(11):695–711. 10.1136/BJSPORTS-2023-106898. 37316210 10.1136/bjsports-2023-106898

[4] King D, Brughelli M, Hume P, Gissane C. Assessment, management and knowledge of sport-related concussion: systematic review. Sports Med. 2014;44(4):449–71. 10.1007/S40279-013-0134-X. 24403125 10.1007/s40279-013-0134-x

[5] Kieffer EE, Begonia MT, Tyson AM, Rowson S. A two-phased approach to quantifying head impact sensor accuracy: in-laboratory and on-field assessments. Ann Biomed Eng. 2020;48(11):2613–25. 10.1007/S10439-020-02647-1. 33051745 10.1007/s10439-020-02647-1

[6] O’Connor KL, Rowson S, Duma SM, Broglio SP. Head-impact–measurement devices: a systematic review. J Athl Train. 2017;52(3):206–27. 10.4085/1062-6050.52.2.05. 28387553 10.4085/1062-6050.52.2.05 PMC5384819

[7] Nguyen JVK, Brennan JH, Mitra B, Willmott C. Frequency and magnitude of game-related head impacts in male contact sports athletes: a systematic review and meta-analysis. Sports Med. 2019;49(10):1575–83. 10.1007/S40279-019-01135-4. 31175636 10.1007/s40279-019-01135-4

[8] Brennan JH, Mitra B, Synnot A, et al. Accelerometers for the assessment of concussion in male athletes: a systematic review and meta-analysis. Sports Med. 2017;47(3):469–78. 10.1007/S40279-016-0582-1. 27402455 10.1007/s40279-016-0582-1

[9] Patton DA. A review of instrumented equipment to investigate head impacts in sport. Appl Bionics Biomech. 2016. 10.1155/2016/7049743. 27594780 10.1155/2016/7049743 PMC4993933

[10] King D, Hume P, Gissane C, Brughelli M, Clark T. The influence of head impact threshold for reporting data in contact and collision sports: systematic review and original data analysis. Sports Med. 2015;46(2):151–69. 10.1007/S40279-015-0423-7. 10.1007/s40279-015-0423-7 26545363

[11] Funk JR, McIntosh AS, Withnall C, Wonnacott M, Jadischke R. Best practices for conducting physical reconstructions of head impacts in sport. Ann Biomed Eng. 2022;50(11):1409–22. 10.1007/S10439-022-03024-W/FIGURES/1. 35876938 10.1007/s10439-022-03024-w

[12] Cortes N, Lincoln AE, Myer GD, et al. Video analysis verification of head impact events measured by wearable sensors. Am J Sports Med. 2017;45(10):2379–87. 10.1177/0363546517706703. 28541813 10.1177/0363546517706703

[13] Kuo C, Wu L, Loza J, Senif D, Anderson SC, Camarillo DB. Comparison of video-based and sensor-based head impact exposure. PLoS ONE. 2018;13(6): e0199238. 10.1371/JOURNAL.PONE.0199238. 29920559 10.1371/journal.pone.0199238 PMC6007917

[14] Patton DA, Huber CM, Jain D, et al. Head impact sensor studies in sports: a systematic review of exposure confirmation methods. Ann Biomed Eng. 2020;48(11):2497–507. 10.1007/S10439-020-02642-6/TABLES/2. 33051746 10.1007/s10439-020-02642-6 PMC7674240

[15] Rowson B, Duma SM. A review of on-field investigations into the biomechanics of concussion in football and translation to head injury mitigation strategies. Ann Biomed Eng. 2020;48(12):2734–50. 10.1007/S10439-020-02684-W. 33200263 10.1007/s10439-020-02684-w

[16] Basinas I, McElvenny DM, Pearce N, Gallo V, Cherrie JW. A systematic review of head impacts and acceleration associated with soccer. Int J Environ Res Public Health. 2022;19(9):5488. 10.3390/IJERPH19095488/S1. 35564889 10.3390/ijerph19095488 PMC9100160

[17] Worsey MTO, Espinosa HG, Shepherd JB, Thiel DV. Inertial sensors for performance analysis in combat sports: a systematic review. Sports. 2019;7(1):28. 10.3390/SPORTS7010028. 30669590 10.3390/sports7010028 PMC6359075

[18] Le Flao E, Siegmund GP, Borotkanics R. Head impact research using inertial sensors in sport: a systematic review of methods, demographics, and factors contributing to exposure. Sports Med. 2021;52(3):481–504. 10.1007/S40279-021-01574-Y. 34677820 10.1007/s40279-021-01574-y

[19] Page MJ, McKenzie JE, Bossuyt PM, The PRISMA, et al. statement: an updated guideline for reporting systematic reviews. BMJ. 2020;2021:372. 10.1136/BMJ.N71. 10.1136/bmj.n71 PMC8005924 33782057

[20] Brooke BS, Schwartz TA, Pawlik TM. MOOSE reporting guidelines for meta-analyses of observational studies. JAMA Surg. 2021;156(8):787–8. 10.1001/JAMASURG.2021.0522. 33825847 10.1001/jamasurg.2021.0522

[21] Wohlin C, Kalinowski M, Romero Felizardo K, Mendes E. Successful combination of database search and snowballing for identification of primary studies in systematic literature studies. Inf Softw Technol. 2022;147: 106908. 10.1016/J.INFSOF.2022.106908.

[22] Kohl C, McIntosh EJ, Unger S, et al. Online tools supporting the conduct and reporting of systematic reviews and systematic maps: a case study on CADIMA and review of existing tools. Environ Evid. 2018;7(1):1–17. 10.1186/S13750-018-0115-5/TABLES/3.

[23] Hozo SP, Djulbegovic B, Hozo I. Estimating the mean and variance from the median, range, and the size of a sample. BMC Med Res Methodol. 2005;5(1):1–10. 10.1186/1471-2288-5-13/TABLES/3. 15840177 10.1186/1471-2288-5-13 PMC1097734

[24] Higgins JPT, Thomas J, Chandler J, et al. Cochrane Handbook for Systematic Reviews of Interventions version 6.4 (updated August 2023). Cochrane. 2023. http://www.training.cochrane.org/handbook. Accessed 12 Feb 2024.

[25] Rohatgi A. WebPlotDigitizer (version 4.2) [Computer software]. Pacifica, California, USA [updated August, 2021]. Published online 2021. https://www.automerisio/WebPlotDigitizer. Accessed 12 Feb 2024.

[26] Mamikutty R, Aly AS, Marhazlinda J. Selecting risk of bias tools for observational studies for a systematic review of anthropometric measurements and dental caries among children. Int J Environ Res Public Health. 2021;18(16):8623. 10.3390/IJERPH18168623/S1. 34444374 10.3390/ijerph18168623 PMC8391268

[27] Zeng X, Zhang Y, Kwong JSW, et al. The methodological quality assessment tools for preclinical and clinical studies, systematic review and meta-analysis, and clinical practice guideline: a systematic review. J Evid Based Med. 2015;8(1):2–10. 10.1111/JEBM.12141. 25594108 10.1111/jebm.12141

[28] Moskalewicz A, Oremus M. No clear choice between Newcastle-Ottawa Scale and appraisal tool for cross-sectional studies to assess methodological quality in cross-sectional studies of health-related quality of life and breast cancer. J Clin Epidemiol. 2020;120:94–103. 10.1016/J.JCLINEPI.2019.12.013. 31866469 10.1016/j.jclinepi.2019.12.013

[29] Singh M, Kaur J, Singh S, et al. Comparison of Newcastle Ottawa scale (NOS) and Agency for Health Research and Quality (AHRQ) as risk of bias assessment tools for cohort studies. Filtering the information overload for better decisions. In: 23rd Cochrane Colloquium; 2015.

[30] Maier M, VanderWeele TJ, Mathur MB. Using selection models to assess sensitivity to publication bias: a tutorial and call for more routine use. Campbell Syst Rev. 2022. 10.1002/CL2.1256. 36909879 10.1002/cl2.1256 PMC9247867

[31] Hedges LV, Vevea JL. Estimating effect size under publication bias: small sample properties and robustness of a random effects selection model. J Educ Behav Stat. 1996;21(4):299–332. 10.3102/10769986021004299.

[32] Borenstein M, Hedges LV, Higgins JPT, Rothstein HR. Introduction to meta-analysis. New York: Wiley; 2021.

[33] Higgins JPT, Thompson SG, Deeks JJ, Altman DG. Measuring inconsistency in meta-analyses. BMJ. 2003;327(7414):557–60. 10.1136/BMJ.327.7414.557. 12958120 10.1136/bmj.327.7414.557 PMC192859

[34] Haidich AB. Meta-analysis in medical research. Hippokratia. 2010;14(Suppl 1):29. 21487488 PMC3049418

[35] Thompson SG, Higgins JPT. How should meta-regression analyses be undertaken and interpreted? Stat Med. 2002;21(11):1559–73. 10.1002/SIM.1187. 12111920 10.1002/sim.1187

[36] Harrer M, Cuijpers P, Furukawa TA, Ebert DD. Doing meta-analysis with R: a hands-on guide. Doing meta-analysis with R. Published online September 14, 2021. 10.1201/9781003107347.

[37] Bussey MD, Salmon D, Romanchuk J, et al. Head acceleration events in male community rugby players: an observational cohort study across four playing grades, from under-13 to senior men. Sports Med. 2023. 10.1007/s40279-023-01923-z. 37676621 10.1007/s40279-023-01923-z PMC10933157

[38] Bussey MD, Davidson P, Salmon D, Romanchuk J, Tong D, Sole G. Influence of the frame of reference on head acceleration events recorded by instrumented mouthguards in community rugby players. BMJ Open Sport Exerc Med. 2022. 10.1136/bmjsem-2022-001365. 36249488 10.1136/bmjsem-2022-001365 PMC9557771

[39] Carey L, Stanwell P, Terry DP, et al. Verifying head impacts recorded by a wearable sensor using video footage in rugby league: a preliminary study. Sports Med Open. 2019. 10.1186/s40798-019-0182-3. 30874938 10.1186/s40798-019-0182-3 PMC6419663

[40] Carey L, Terry DP, McIntosh AS, Stanwell P, Iverson GL, Gardner AJ. Video analysis and verification of direct head impacts recorded by wearable sensors in junior rugby league players. Sports Med Open. 2021. 10.1186/s40798-021-00353-3. 34529180 10.1186/s40798-021-00353-3 PMC8446122

[41] Chan EYK, Jones C, Austin K, Loosemore M, Ghajari M. 791 BO14—mixed martial arts vs rugby: which sport produces larger deformation of brain white matter? Br J Sports Med. 2024;58(Suppl 2):A50–1. 10.1136/BJSPORTS-2024-IOC.90.

[42] Field B, Waddington G, McKune A, Goecke R, Gardner AJ. Validation of an instrumented mouthguard in rugby union-a pilot study comparing impact sensor technology to video analysis. Front Sports Act Living. 2023. 10.3389/FSPOR.2023.1230202. 38053522 10.3389/fspor.2023.1230202 PMC10694248

[43] Kieffer EE, Rowson S. Implementing head impact sensors in collegiate men’s and women’s rugby: successes and challenges in characterizing concussion. Virginia Tech. Published online January 29, 2022. http://hdl.handle.net/10919/108015. Accessed 15 Feb 2024.

[44] King D, Hume PA, Brughelli M, Gissane C. Instrumented mouthguard acceleration analyses for head impacts in amateur rugby union players over a season of matches. Am J Sports Med. 2015;43(3):614–24. 10.1177/0363546514560876. 25535096 10.1177/0363546514560876

[45] Langevin TL, Antonoff D, Renodin C, et al. Head impact exposures in women’s collegiate rugby. Physician Sportsmed. 2021;49(1):68–73. 10.1080/00913847.2020.1770568. 10.1080/00913847.2020.1770568 32419585

[46] Manning KY, Brooks JS, Dickey JP, et al. Longitudinal changes of brain microstructure and function in nonconcussed female rugby players. Neurology. 2020;95(4):E402–12. 10.1212/WNL.0000000000009821. 32554762 10.1212/WNL.0000000000009821 PMC7455316

[47] Tooby J, Weaving D, Al-Dawoud M, Tierney G. Quantification of head acceleration events in rugby league: an instrumented mouthguard and video analysis pilot study. Sensors. 2022. 10.3390/s22020584. 35062545 10.3390/s22020584 PMC8781372

[48] Waldron M, Jones C, Melotti L, Brown R, Kilduff LP. Collision monitoring in elite male rugby union using a new instrumented mouth-guard. J Sport Exerc Sci. 2021;5(3):179–87.

[49] Williams EMP, Petrie FJ, Pennington TN, et al. Sex differences in neck strength and head impact kinematics in university rugby union players. Eur J Sport Sci. 2022;22(11):1649–58. 10.1080/17461391.2021.1973573. 34463209 10.1080/17461391.2021.1973573

[50] Edwards S, Lee R, Fuller G, et al. 3D Biomechanics of rugby tackle techniques to inform future rugby research practice: a systematic review. Sports Med Open. 2021;7(1):1–20. 10.1186/S40798-021-00322-W/FIGURES/3. 34097146 10.1186/s40798-021-00322-w PMC8184906

[51] Geeson-Brown T, Jones B, Till K, Chantler S, Deighton K. Body composition differences by age and playing standard in male rugby union and rugby league: a systematic review and meta-analysis. J Sports Sci. 2020;38(19):2161–76. 10.1080/02640414.2020.1775990. 32546054 10.1080/02640414.2020.1775990

[52] Tooby J, Till K, Gardner A, et al. When to pull the trigger: conceptual considerations for approximating head acceleration events using instrumented mouthguards. Sports Med. 2024. 10.1007/S40279-024-02012-5. 38460080 10.1007/s40279-024-02012-5 PMC11239719

[53] Jones B, Tooby J, Weaving D, et al. Ready for impact? A validity and feasibility study of instrumented mouthguards (iMGs). Br J Sports Med. 2022;56(20):1171–9. 10.1136/BJSPORTS-2022-105523. 10.1136/bjsports-2022-105523 35879022

[54] Jones CM, Austin K, Augustus SN, et al. An instrumented mouthguard for real-time measurement of head kinematics under a large range of sport specific accelerations. Sensors. 2023;23(16):7068. 10.3390/S23167068. 37631606 10.3390/s23167068 PMC10457941

[55] Jones CM, Brown MR. Validation of an instrumented mouthguard. MedRxiv. 2022. 10.1101/2022.03.02.22271563.35982668

[56] Campbell KR, Warnica MJ, Levine IC, et al. Laboratory vvaluation of the gForce Tracker™, a head impact kinematic measuring device for use in football helmets. Ann Biomed Eng. 2016;44(4):1246–56. 10.1007/S10439-015-1391-7/TABLES/3. 26198174 10.1007/s10439-015-1391-7

[57] Edriss S, Romagnoli C, Caprioli L, et al. The role of emergent technologies in the dynamic and kinematic assessment of human movement in sport and clinical applications. Appl Sci. 2024;14(3):1012. 10.3390/APP14031012.

[58] Tierney G, Rowson S, Gellner R, et al. Head Exposure to Acceleration Database in Sport (HEADSport): a kinematic signal processing method to enable instrumented mouthguard (iMG) field-based inter-study comparisons. BMJ Open Sport Exerc Med. 2024. 10.1136/BMJSEM-2023-001758. 38304714 10.1136/bmjsem-2023-001758 PMC10831454

[59] Hunter LE, Branch CA, Lipton ML. The neurobiological effects of repetitive head impacts in collision sports. Neurobiol Dis. 2019;123:122–6. 10.1016/J.NBD.2018.06.016. 29936233 10.1016/j.nbd.2018.06.016 PMC6453577

[60] Hack L, Singh B, Binkofski F, Helmich I. Repetitive subconcussive head impacts in sports and their impact on brain anatomy and function. Int J Sports Med. 2024;45(12):871–83. 10.1055/A-2342-3604/ID/RIJSM-01-2024-10444-0013/BIB. 38857880 10.1055/a-2342-3604

[61] Sundaram V, Sundar V, Pearce AJ. Biomechanical characteristics of concussive and sub-concussive impacts in youth sports athletes: a systematic review and meta-analysis. J Sports Sci. 2023;41(7):631–45. 10.1080/02640414.2023.2231317. 37393593 10.1080/02640414.2023.2231317

[62] King D, Hume P, Gissane C, Clark T. Semi-professional rugby league players have higher concussion risk than professional or amateur participants: a pooled analysis. Sports Med. 2017;47(2):197–205. 10.1007/S40279-016-0576-Z/TABLES/4. 27351803 10.1007/s40279-016-0576-z

[63] Broglio SP, Surma T, Ashton-Miller JA. High school and collegiate football athlete concussions: a biomechanical review. Ann Biomed Eng. 2012;40(1):37–46. 10.1007/S10439-011-0396-0/TABLES/1. 21994058 10.1007/s10439-011-0396-0

[64] Pankow MP, Syrydiuk RA, Kolstad AT, et al. Head games: a systematic review and meta-analysis examining concussion and head impact incidence rates, modifiable risk factors, and prevention strategies in youth tackle football. Sports Med. 2022;52(6):1259–72. 10.1007/S40279-021-01609-4/TABLES/2. 34894348 10.1007/s40279-021-01609-4

