# Correction: Unlocking the Impact: A Systematic Review and Meta-Analysis of Biomechanical Insights into Rugby Head Impacts Using Wearable Sensor Technology

**DOI:** 10.1007/s40279-025-02320-4

**Published:** 2025-09-18

**Authors:** Luis De Sousa-De Sousa, Hugo G. Espinosa, José Luis Maté-Muñoz, Roberto Murias-Lozano, Mario Iglesias Muñiz, Francisco Javier San Sebastián Obregón, Cristian Solís-Mencía, Pablo García-Fernández

**Affiliations:** 1 Department of Radiology, Rehabilitation and Physiotherapy, Faculty of Nursing, Physiotherapy and Podiatry, Complutense University of Madrid, 28040 Madrid, Spain; 2 School of Engineering and Built Environment, Griffith University, Brisbane, QLD 4111 Australia; 3 Department of Physiotherapy, Camilo José Cela University, Villafranca del Castillo, 28692 Madrid, Spain; 4 Centro Médico-Quirúrgico Olympia, P.º de la Castellana, 259, Fuencarral-El Pardo, 28046 Madrid, Spain; 5 Spanish Rugby Federation, 28008 Madrid, Spain; 6 Department of Medicine, Faculty of Health Sciences, University of Deusto, Bilbao, Bizkaia Spain; 7 Grupo InPhysio, Instituto de Investigación Sanitaria del Hospital Clínico San Carlos (IdISSC), Madrid, Spain

**Correction: Sports Medicine** 10.1007/s40279-025-02228-z

The article [A Systematic Review and Meta-Analysis of Biomechanical Insights into Rugby Head Impacts Using Wearable Sensor Technology], written by [Luis De Sousa‑De Sousa · Hugo G. Espinosa2 · José Luis Maté‑Muñoz 1 · Roberto Murias‑Lozano4 · Mario Iglesias Muñiz · Francisco Javier San Sebastián Obregón · Cristian Solís‑Mencía · Pablo García‑Fernández], was originally published Online First without Open Access. After publication in volume [55], issue [8] the authors decided to opt for Open Choice and to make the article an Open Access publication. Therefore, the copyright of the article has been changed to ©[The Author(s)] [2025] and this article is licensed under a Creative Commons Attribution 4.0 International License, which permits use, sharing, adaptation, distribution and reproduction in any medium or format, as long as you give appropriate credit to the original author(s) and the source, provide a link to the Creative Commons licence, and indicate if changes were made. The images or other third party material in this article are included in the article’s Creative Commons licence, unless indicated otherwise in a credit line to the material. If material is not included in the article’s Creative Commons licence and your intended use is not permitted by statutory regulation or exceeds the permitted use, you will need to obtain permission directly from the copyright holder. To view a copy of this licence, visit http://creativecommons.org/licenses/by/4.0/].

The original article has been corrected.

**Funding** Open Access provided thanks to the CRUE-CSIC agreement with Springer Nature.

