# Supplementary material for: Unlocking the Impact: A Systematic Review and Meta-Analysis of Biomechanical Insights into Rugby Head Impacts Using Wearable Sensor Technology
Source: Sports Med. 2025 May 3;55(8):1903–21. doi: 10.1007/s40279-025-02228-z (PMC12460405; doi:10.1007/s40279-025-02228-z)
Supplement: Supplementary file 1 — Supplementary file1 (DOCX 27 KB) [file 40279_2025_2228_MOESM1_ESM.docx]

**PUBMED – NO RESTRICTIONS**

**#1** 'rugby, union play' OR 'play rugby, union' OR 'union play rugbies' OR 'union play rugby' OR 'rugby, league play' OR 'league play rugby' OR 'play rugby, league' OR 'rugby union' OR 'rugby'/exp OR 'rugby'

**#2** 'athletic injuries' OR 'concussion' OR 'sports concussion' OR 'sports-related concussion' OR 'brain concussion' OR 'brain injury' OR 'brain injuries' OR 'mild traumatic brain injury' OR 'mtbi' OR 'traumatic brain injury' OR 'tbi' OR 'craniocerebral trauma' OR 'head injury' OR 'brain damage' OR 'athlet* injuri*' OR ('athlet*' AND 'statistics') OR ('athlet*' AND 'numerical data') OR ('athlet* injur*' AND 'epidemiology') OR ('athlet* injur*' AND 'etiology') OR ('athlet* injur*' AND 'control')

**#3** 'head acceleration*' OR 'accelerometer' OR 'gyroscope' OR 'wearable sensor*' OR 'wearable head sensor' OR 'instrumented mouthguard' OR 'headgear' OR 'instrumented headgear' OR 'instrumented helmet' OR 'instrumented skin patch'

**#4** #1 AND #2 AND #3

**#5** 'rugby'/exp AND 'brain concussion'/exp AND 'biomechanics'/exp AND 'apparatus and instruments'/exp

**#6** #4 OR #5

**TOTAL RETRIEVED: 228**

**EMBASE – NO RESTRICTIONS**

**#1** 'rugby, union play' OR 'play rugby, union' OR 'union play rugbies' OR 'union play rugby' OR 'rugby, league play' OR 'league play rugby' OR 'play rugby, league' OR 'rugby union' OR 'rugby'/exp OR 'rugby'

**#2** 'athletic injuries' OR 'concussion' OR 'sports concussion' OR 'sports-related concussion' OR 'brain concussion' OR 'brain injury' OR 'brain injuries' OR 'mild traumatic brain injury' OR 'mtbi' OR 'traumatic brain injury' OR 'tbi' OR 'craniocerebral trauma' OR 'head injury' OR 'brain damage' OR 'athlet* injuri*' OR ('athlet*' AND 'statistics') OR ('athlet*' AND 'numerical data') OR ('athlet* injur*' AND 'epidemiology') OR ('athlet* injur*' AND 'etiology') OR ('athlet* injur*' AND 'control')

**#3** 'head acceleration*' OR 'accelerometer' OR 'gyroscope' OR 'wearable sensor*' OR 'wearable head sensor' OR 'instrumented mouthguard' OR 'headgear' OR 'instrumented headgear' OR 'instrumented helmet' OR 'instrumented skin patch'

**#4** #1 AND #2 AND #3

**#5** 'rugby'/exp AND 'brain concussion'/exp AND 'biomechanics'/exp AND 'apparatus and instruments'/exp

**#6** #4 OR #5

**TOTAL RETRIEVED: 75**

**WEB OF SCIENCE – NO RESTRICTIONS (SEARCHED BY TOPIC)**

**#1** 'rugby, union play' OR 'play rugby, union' OR 'union play rugbies' OR 'union play rugby' OR 'rugby, league play' OR 'league play rugby' OR 'play rugby, league' OR 'rugby union' OR 'rugby'/exp OR 'rugby'

**#2** 'athletic injuries' OR 'concussion' OR 'sports concussion' OR 'sports-related concussion' OR 'brain concussion' OR 'brain injury' OR 'brain injuries' OR 'mild traumatic brain injury' OR 'mtbi' OR 'traumatic brain injury' OR 'tbi' OR 'craniocerebral trauma' OR 'head injury' OR 'brain damage' OR 'athlet* injuri*' OR ('athlet*' AND 'statistics') OR ('athlet*' AND 'numerical data') OR ('athlet* injur*' AND 'epidemiology') OR ('athlet* injur*' AND 'etiology') OR ('athlet* injur*' AND 'control')

**#3**  'head acceleration*' OR 'accelerometer' OR 'gyroscope' OR 'wearable sensor*' OR 'wearable head sensor' OR 'instrumented mouthguard' OR 'headgear' OR 'instrumented headgear' OR 'instrumented helmet' OR 'instrumented skin patch'

**#4** #1 AND #2 AND #3

**TOTAL RETRIEVED: 118**

**SCOPUS – NO RESTRICTIONS (SEARCHED BY ARTICLE TITLE, ABSTRACT, KEYWORDS))**

**#1** 'rugby, union play' OR 'play rugby, union' OR 'union play rugbies' OR 'union play rugby' OR 'rugby, league play' OR 'league play rugby' OR 'play rugby, league' OR 'rugby union' OR 'rugby'/exp OR 'rugby'

**#2** 'athletic injuries' OR 'concussion' OR 'sports concussion' OR 'sports-related concussion' OR 'brain concussion' OR 'brain injury' OR 'brain injuries' OR 'mild traumatic brain injury' OR 'mtbi' OR 'traumatic brain injury' OR 'tbi' OR 'craniocerebral trauma' OR 'head injury' OR 'brain damage' OR 'athlet* injuri*' OR ('athlet*' AND 'statistics') OR ('athlet*' AND 'numerical data') OR ('athlet* injur*' AND 'epidemiology') OR ('athlet* injur*' AND 'etiology') OR ('athlet* injur*' AND 'control')

**#3** 'head acceleration*' OR 'accelerometer' OR 'gyroscope' OR 'wearable sensor*' OR 'wearable head sensor' OR 'instrumented mouthguard' OR 'headgear' OR 'instrumented headgear' OR 'instrumented helmet' OR 'instrumented skin patch'

**#4** #1 AND #2 AND #3

**TOTAL RETRIEVED: 85**

**SPORTDISCUS – NO RESTRICTIONS (ALL FIELDS)**

**#1** 'rugby, union play' OR 'play rugby, union' OR 'union play rugbies' OR 'union play rugby' OR 'rugby, league play' OR 'league play rugby' OR 'play rugby, league' OR 'rugby union' OR 'rugby'/exp OR 'rugby'

**#2** 'athletic injuries' OR 'concussion' OR 'sports concussion' OR 'sports-related concussion' OR 'brain concussion' OR 'brain injury' OR 'brain injuries' OR 'mild traumatic brain injury' OR 'mtbi' OR 'traumatic brain injury' OR 'tbi' OR 'craniocerebral trauma' OR 'head injury' OR 'brain damage' OR 'athlet* injuri*' OR ('athlet*' AND 'statistics') OR ('athlet*' AND 'numerical data') OR ('athlet* injur*' AND 'epidemiology') OR ('athlet* injur*' AND 'etiology') OR ('athlet* injur*' AND 'control')

**#3** 'head acceleration*' OR 'accelerometer' OR 'gyroscope' OR 'wearable sensor*' OR 'wearable head sensor' OR 'instrumented mouthguard' OR 'headgear' OR 'instrumented headgear' OR 'instrumented helmet' OR 'instrumented skin patch'

**#4** #1 AND #2 AND #3

**TOTAL RETRIEVED: 31**

**CINHAL – NO RESTRICTIONS**

**#1** 'rugby, union play' OR 'play rugby, union' OR 'union play rugbies' OR 'union play rugby' OR 'rugby, league play' OR 'league play rugby' OR 'play rugby, league' OR 'rugby union' OR 'rugby'/exp OR 'rugby'

**#2** 'athletic injuries' OR 'concussion' OR 'sports concussion' OR 'sports-related concussion' OR 'brain concussion' OR 'brain injury' OR 'brain injuries' OR 'mild traumatic brain injury' OR 'mtbi' OR 'traumatic brain injury' OR 'tbi' OR 'craniocerebral trauma' OR 'head injury' OR 'brain damage' OR 'athlet* injuri*' OR ('athlet*' AND 'statistics') OR ('athlet*' AND 'numerical data') OR ('athlet* injur*' AND 'epidemiology') OR ('athlet* injur*' AND 'etiology') OR ('athlet* injur*' AND 'control')

**#3** 'head acceleration*' OR 'accelerometer' OR 'gyroscope' OR 'wearable sensor*' OR 'wearable head sensor' OR 'instrumented mouthguard' OR 'headgear' OR 'instrumented headgear' OR 'instrumented helmet' OR 'instrumented skin patch'

**#4** #1 AND #2 AND #3

**TOTAL RETRIEVED: 53**

**PSYCINFO – NO RESTRICTIONS**

**#1** 'rugby, union play' OR 'play rugby, union' OR 'union play rugbies' OR 'union play rugby' OR 'rugby, league play' OR 'league play rugby' OR 'play rugby, league' OR 'rugby union' OR 'rugby'/exp OR 'rugby'

**#2** 'athletic injuries' OR 'concussion' OR 'sports concussion' OR 'sports-related concussion' OR 'brain concussion' OR 'brain injury' OR 'brain injuries' OR 'mild traumatic brain injury' OR 'mtbi' OR 'traumatic brain injury' OR 'tbi' OR 'craniocerebral trauma' OR 'head injury' OR 'brain damage' OR 'athlet* injuri*' OR ('athlet*' AND 'statistics') OR ('athlet*' AND 'numerical data') OR ('athlet* injur*' AND 'epidemiology') OR ('athlet* injur*' AND 'etiology') OR ('athlet* injur*' AND 'control')

**#3** 'head acceleration*' OR 'accelerometer' OR 'gyroscope' OR 'wearable sensor*' OR 'wearable head sensor' OR 'instrumented mouthguard' OR 'headgear' OR 'instrumented headgear' OR 'instrumented helmet' OR 'instrumented skin patch'

**#4** #1 AND #2 AND #3

**TOTAL RETRIEVED: 6**

**TOTAL SUM OF ALL DATA BASES: 596**
