# Supplementary material for: Unlocking the Impact: A Systematic Review and Meta-Analysis of Biomechanical Insights into Rugby Head Impacts Using Wearable Sensor Technology
Source: Sports Med. 2025 May 3;55(8):1903–21. doi: 10.1007/s40279-025-02228-z (PMC12460405; doi:10.1007/s40279-025-02228-z)
Supplement: Supplementary file 2 — Supplementary file2 (DOCX 22 KB) [file 40279_2025_2228_MOESM2_ESM.docx]

**Appendix 1:**

**NEWCASTLE-OTTAWA QUALITY ASSESSMENT SCALE**

**(Adapted for Cross-Sectional Studies - Head Impacts in Rugby Players)**

**SELECTION (5/5):**

- Representativeness Of The Cases:

a) Truly representative of the rugby players (consecutive or random sampling). ☆

b) Somewhat representative of the average rugby players (non-random sampling). ☆

c) Selected demographic group of users. (0 stars)

d) No description of the sampling strategy. (0 stars)

- Sample Size:

a) Justified and satisfactory (considering the nature of your study). ☆

b) Not justified. (0 stars)

- Non-Response Rate:

a) The response rate is satisfactory (considering the nature of your study). ☆

b) The response rate is unsatisfactory (<95%), or no description. (0 stars)

- Ascertainment of Head Impacts

a) Validated tool for head impact measurement. ☆☆

b) Non-validated tool, but the tool is available or described. ☆

c) No description of the measurement tool. (0 stars)

**COMPARABILITY (1/1):**

- Investigation of Potential Confounders:

a) The study investigates potential confounders. ☆

b) The study does not investigate potential confounders. (0 stars)

**OUTCOME (3/3):**

- Assessment of Head Impacts:

a) Independent blind assessment. ☆☆

b) Record linkage. ☆☆

c) Self-report. ☆

d) No description. (0 stars)

- Statistical Test:

a) The statistical test used is clearly described and appropriate. ☆

b) The statistical test is not appropriate, not described, or incomplete. (0 stars)

Interpretation:

≥7 points were considered as “good”, 2 to 6 points were considered as “fair”, and ≤1 point was considered as “poor” quality.
