# Supplementary material for: Unlocking the Impact: A Systematic Review and Meta-Analysis of Biomechanical Insights into Rugby Head Impacts Using Wearable Sensor Technology
Source: Sports Med. 2025 May 3;55(8):1903–21. doi: 10.1007/s40279-025-02228-z (PMC12460405; doi:10.1007/s40279-025-02228-z)
Supplement: Supplementary file 3 — Supplementary file3 (DOCX 41 KB) [file 40279_2025_2228_MOESM3_ESM.docx]

| **Rugby Branch** | **Threshold** | **Study** | **Data Reports** | **Report details** | **Mean Head Impact PLA (SE)** |
| --- | --- | --- | --- | --- | --- |
| **RU** | **5g>** | **Bussey et al. (36)** | 8 | M U13 (Trainings) | 12.90 (0.04) |
|  |  |  |  | M U15 (Trainings) | 12.00 (0.05) |
|  |  |  |  | M U19 (Trainings) | 12.60 (0.07) |
|  |  |  |  | M S (Trainings) | 12.10 (0.08) |
|  |  |  |  | M U13 (Matches) | 11,81 (0.06) |
|  |  |  |  | M U15 (Matches) | 13,42 (0.06) |
|  |  |  |  | M U19 (Matches) | 13,36 (0.07) |
|  |  |  |  | S (Matches) | 13,40 (0.11) |
|  |  | **Bussey et al. (37)** | 1 | M (Matches) | 17,10 (0,32) |
|  |  | **Kieffer et al. (43)** | 2 | F (Combined Trainings/matches) | 15,30 (1.60) |
|  |  |  |  | M (Combined Trainings/matches) | 14,60 (0.79) |
|  | **10g>** | **Chan et al. (40)** | 1 | M (matches) | 18,80 (1.18) |
|  |  | **Field et al. (41)** | 1 | M (matches) | 23,05 (0.20) |
|  |  | **King et al. (45)** | 1 | M (matches) | 22,20 (0.11) |
|  |  | **Waldron et al. (51)** | 1 | M (matches) | 16,80 (0.08) |
|  |  | **Williams et al. (52)** | 2 | F (matches) | 11,60 (0.79) |
|  |  |  |  | M (matches) | 12,50 (0.49) |
|  | **15g>** | **Langevin et al. (48)** | 2 | F (Trainings) | 29,90 (0.31) |
|  |  |  |  | F (Matches) | 31,90 (0.30) |
|  |  | **Manning et al. (49)** | 1 | F (Combined Trainings/matches) | 22,90 (0.40) |
| **RL** | **5g>** | **Tooby et al. (50)** | 1 | M (matches) | 7,30 (0.03) |
|  | **20g>** | **Carey et al. (35)** | 1 | M (matches) | 34,20 (0.77) |
|  |  | **Carey et al. (36)** | 1 | M (N/R) | 34,10 (0.67) |

*F: Female; M: Male; N/R: Not reported;RL: Rugby League; RU: Rugby Union S: Senior; U13: Under thirteen; U15: Under fifteen; U19: Under nineteen.*

***Table 7****: Study Contributions to PLA Pooled Data Presented in Forest Plot (Figure 2).*
